# Resveratrol potentiates chemotherapeutic efficacy of olaparib in MCF7 human breast cancer cells by inducing apoptosis

**DOI:** 10.1007/s11845-025-04240-8

**Published:** 2026-01-10

**Authors:** Mehmet Kadir Erdoğan, Ramazan Gundogdu, Yusuf Toy, Esra Polat, Can Ali Agca, Aydın Sever, Burak Tüzün

**Affiliations:** 1 Department of Molecular Biology and Genetics, Bingol University, 12000 Bingol, Türkiye; 2 Department of Pharmacy Services, Vocational School of Health Services, Bingol University, 12000 Bingol, Türkiye; 3 Department of Biology, Institute of Science, Bingol University, 12000 Bingol, Türkiye; 4 Department of Molecular Biology and Genetics, Institute of Science, Bingol University, Bingol, 12000 Türkiye; 5 Plant and Animal Production Department, Technical Sciences Vocational School of Sivas, Sivas Cumhuriyet University, Sivas, 58140 Türkiye

**Keywords:** Resveratrol, PARP inhibition, MCF7, Personalised medicine

## Abstract

**Background:**

Resveratrol is a natural polyphenolic compound found in grapes, berries, and peanuts and is well recognized for its cancer-preventive and anticancer properties. Poly(ADP-ribose) polymerase (PARP) inhibitors, such as olaparib, represent a novel class of targeted anticancer agents that impair DNA single-strand break repair, ultimately leading to genomic instability and cell death. This study aimed to investigate whether resveratrol enhances the anticancer efficacy of the PARP inhibitor olaparib in breast cancer cells.

**Methods:**

MCF7 breast cancer cells were treated with resveratrol and olaparib, alone or in combination. Cell viability was assessed using WST- 1 and crystal-violet assays at 24, 48, 72, and 96 h to determine IC₅₀ values and combination index (CI) values. Anticancer effects were further evaluated using clonogenic survival, colony formation, and wound-healing assays. DNA damage and apoptosis were analyzed by DNA ladder assays, acridine orange/ethidium bromide (AO/EB) staining, and Western blotting. In silico analyses were performed using the Gaussian package at B3LYP, HF, and M062x levels with 6–31 g, 6–31++g, and 6–31++g(d,p) basis sets. Molecular docking against breast cancer–related proteins (PDB ID: 1A52 and 1JNX) and ADME/T property predictions were also conducted.

**Results:**

Resveratrol exhibited time-dependent cytotoxicity with IC₅₀ values of 124.55 ± 4.23, 98.98 ± 4.37, 82.45 ± 1.50, and 76.86 ± 2.76 μM at 24, 48, 72, and 96 h, respectively. Olaparib IC₅₀ values were 9.60 ± 0.68, 7.08 ± 0.31, 5.24 ± 0.23, and 4.85 ± 0.26 μM at the same time points. Synergistic interactions between resveratrol and olaparib were observed at 48, 72, and 96 h, with CI values of 0.91, 0.67, and 0.62, respectively. Functional assays demonstrated that resveratrol significantly potentiated the inhibitory effects of olaparib on clonogenicity, colony formation, and cell migration. Apoptosis-related assays confirmed enhanced DNA damage and apoptotic cell death in the combination treatment group. Computational analyses supported favorable interactions of the resveratrol–olaparib combination with breast cancer–related protein targets and acceptable ADME/T properties.

**Conclusion:**

These findings demonstrate that resveratrol synergistically enhances the anticancer activity of olaparib by reducing cell viability, increasing DNA damage, and promoting apoptosis in MCF7 breast cancer cells. The combination of resveratrol with PARP inhibition may represent a promising therapeutic strategy for improving breast cancer treatment outcomes.

## Introduction

 Natural products are compounds found in abundant natural sources like plants, possessing biological effects. Many therapeutic drugs, such as alkaloids, taxanes, and flavonoids, are derived from these sources. Recently, there has been a growing interest in using natural compounds in chemotherapy to improve the effectiveness of specific anti-cancer drugs. Furthermore, the use of natural compounds like phytochemicals, minerals, and vitamins in cancer treatment has shown promising outcomes against various types of malignant tumours. As a result, natural compounds with strong anti-tumour properties and minimal toxicity in healthy tissues are being explored for their potential synergistic effects with traditional anti-cancer medications [1–3].

Resveratrol (3,5,4’-trans-trihydroxystilbene), with stilbene structure, is a naturally occurring polyphenolic phytoalexin that is widely present in high amounts in many plant sources including grapes, berries, blueberries, pistachios and peanut [4, 5]. An increasing amount of evidence demonstrates resveratrol to have cancer-preventive and anti-cancer activities, enabling it to be considered for cancer treatment [6–9]. Numerous preclinical research have revealed that resveratrol possesses strong cytotoxic activities against various human cancer cells including breast, ovary, prostate, colon and pancreas [7]. Indeed, several in vivo studies have also shown the anti-neoplastic potential of resveratrol in chemically-induced [10, 11] and xenograft animal breast cancer models [12].

It has been reported that resveratrol has an in vitro cytotoxic activities which impede cancer cell growth by inducing apoptosis in MCF7 and MDA-MB-231 breast cancer cell lines via the Akt-caspase-9 pathway [13]. Recent anti-cancer studies mostly focus on deciphering the chemosensitisation potential of resveratrol in a wide-range of cancer cell models, including breast, ovarian and lung cancers [14–19]. Resveratrol has been also demonstrated to enhance the cytotoxic capacity of a widely-used anti-cancer agent, melphalan, in MCF7 and MDA-MB-231 cells through cell cycle arrest [14]. Research to understand the cytotoxic mechanism of resveratrol found that resveratrol inhibits various genes involved in different cellular mechanisms including homologous recombination (HR) DNA repair [20]. Indeed, the same study conducted in MCF7 cells demonstrated that resveratrol significantly reduces protein [20] and mRNA levels [19] of the heterotrimeric MRN complex (Mre11-Nbs1-Rad50), known as a DNA damage sensor that has essential roles in HR [21]. Furthermore, resveratrol has been also shown to sensitise MCF7 breast and HeLa ovarian cancer cells towards chemotherapeutic agents by reducing RAD51 expression, a key recombinase required for an effective HR mechanism [19, 22]. Taken together, these studies indicate that resveratrol may need to be considered in combination with other anti-cancer agents that specifically utilise HR-deficiency in order to improve treatment efficacy, manage off-target toxicity and overcome chemoresistance.

The ultimate goal of personalised cancer research is to identify molecular components that serve as novel pharmacological targets. These targets enable clinicians to specifically target cancer cells, offering the potential for more effective anti-cancer therapy. Additionally, these molecular components can function as predictive biomarkers, allowing clinicians to stratify patients and provide tailored treatment plans for improved outcomes in the clinic. The poly (ADP-ribose) polymerase family consists of 17 proteins which participate in various cellular activities, including DNA damage detection and repair, chromatin remodelling, stress response and apoptosis [23–25]. Of those, PARP1 plays significant roles to detect and manage the repairment of DNA single-strand breaks (SSBs). PARP inhibitors (PARPi) are identified as novel class of targeted therapies that impede the catalytic activity of PARP proteins, leading the accumulation of SSBs in the genome [26–28]. Not only the extensive accumulation of SSBs but also PARP molecules trapped on chromatin induces catastrophic DNA double-strand breaks (DSBs), subjected to be repaired by the HR double-strand break repair mechanism. If these lethal lesions are left unrepaired, they threaten the integrity of the genome, ultimately causing cell death [26, 29]. Although cells with efficient HR mechanism are able to deal with induced DSBs, HR-deficient cancer cells cannot manage the survival upon the overwhelming burden of DSB accumulation [26, 29–31]. BRCA1/2 proteins have important regulatory roles in HR mechanism and the BRCA1/2-mutated, therefore HR-deficient, cancer cells are specifically targeted and eliminated by PARPi molecules developed so far. As a result of synthetic lethal interaction defined between compromised BRCA1/2- and PARP-driven processes, various clinically approved PARPi including olaparib are widely used in the clinic to treat patients with ovarian, breast, pancreatic and prostate cancers [28, 32–38]. Given the relatively low prevalence of *BRCA1/2* deficiency among breast cancer patients and the emergence of de novo or acquired resistance to PARPi, researchers are focused on developing combination treatment strategies to enhance the efficacy of PARPi molecules in HR-proficient cancer cells [39–41].

Breast cancer is the most common type of cancer in women and a major health problem worldwide [42]. Although early diagnosis greatly increases the success of the treatment of the disease, the need for effective and targeted treatment methods in advanced breast cancers is increasing [43]. In this context, understanding cancer cells at the molecular level and identifying specific target proteins play a critical role in the development of new drug candidates [44].

Theoretical calculations are a powerful approach that accelerates the drug design process and reduces costs. Methods such as molecular modeling, virtual screening, molecular docking simulations and quantum chemical calculations help predict the interactions of candidate molecules with target proteins, evaluate binding affinity and minimize possible side effects [45]. Prominent target proteins in breast cancer (such as estrogen receptors, BRC repeat protein, HER2) are examined in detail with these methods, allowing the development of more effective and less toxic drugs. In this way, drug design supported by theoretical calculations offers new and promising options in the treatment of breast cancer [46].

In the present study, we evaluated the combinational effects of resveratrol and PARP inhibitor olaparib in MCF7 breast cancer cells. Our results revealed significantly reduced cell viability, colony survival, clonogenic tumour formation and cellular migration and increased apoptosis in cells treated with olaparib in combination with resveratrol. Consequently, our study suggest that this strategy can be used to promote the sensitisation of breast cancer cells and enhance the effect of current PARPi therapies. Resveratrol, olaparib, and their combinations, including three distinct compounds, were evaluated using the Gaussian software at the B3LYP, HF, and M062 × [47–49] levels using the 6–31 g, 6–31 + + g, and 6–31 + + g(d, p) basis sets. The actions of these three compounds against breast cancer proteins (PDB ID: 1A52 and 1JNX) [50, 51] were subsequently compared. The ADME/T characteristics of the compound were ultimately assessed.

## Materials and methods

### Reagents and antibodies

Resveratrol (#N811.1) was purchased from Carl Roth (Essen, Germany). Olaparib (#HY-10162) was bought from MedChemexpress (Monmouth Junction, NJ, USA). WST-1 cell proliferation and cytotoxicity assay kit (#AR-1159) was obtained from Boster Biological Technology (Pleasanton, CA, USA). Crystal violet (#C0775), neutral red (N4638), acridine orange base (#235474) and ethidium bromide (#E7637) were procured from Sigma-Aldrich (St. Louis MO, USA). Ultra-low gelling temperature agarose (#A2576) was bought from Sigma-Aldrich (St. Louis MO, USA). Dulbecco’s Modified Eagle’s Medium (DMEM, high glucose; #D6429) was purchased from Sigma-Aldrich (St. Louis MO). Heat-inactivated fetal bovine serum (FBS; #S191H) were obtained from Biowest (Nuaillé - France). The following primary antibodies were used in immunoblotting: rabbit monoclonal anti-cleaved-PARP (Asp214, Cell Signaling Technology; #9541; 1:500), mouse monoclonal anti-p53 (Santa Cruz, #sc126; 1:1000), mouse monoclonal anti-p21 (Santa Cruz, #sc6246; 1:1000), rabbit monoclonal anti-γH2AX (Ser139, Cell Signaling Technology, #9718, 1:500). Rat polyclonal anti-tubulin was kindly provided by Dr. Alexander Hergovich (Evotec, France). The following secondary antibodies were employed in immunoblotting: goat anti-rat IgG HRP-conjugated (Invitrogen, #31470; 1:5000), goat anti-mouse IgG HRP-conjugated (Invitrogen, #31430; 1:5000), goat anti-rabbit IgG HRP-conjugated (Invitrogen, #31460; 1:5000).

### Cell culture and cell treatments

MCF7 breast cancer cell line was obtained from Dr. Alexander Hergovich (Evotec, France) and cultured in DMEM supplemented with 10% fetal bovine serum (FBS), 100 U/mL penicillin, 100 µg/mL streptomycin and 2.5 µg/mL amphotericin B (Biowest, #L0010). Cells were maintained at 37 °C in a humidified environment with 5% CO_2_. Exponentially growing cells were seeded in a suitable cell culture plate format and next day, cells were treated with resveratrol and/or olaparib in plating medium as indicated.

### WST1 cytotoxicity assay and calculation of combination index (CI)

The cytotoxic capacity of indicated chemical components applied alone or in combination was assessed by the WST-1 assay, conducted as described [52]. Briefly, 5 × 10E3 cells per well were seeded into a 96-well microplate and incubated overnight at 37 °C with 5% CO_2_. The cells were then treated with indicated concentrations of resveratrol and/or olaparib for 72 h. Next, 10 µL of WST-1 reagent was added to wells and after 2 h of incubation, the colour change was measured at 450 nm as the reference wavelength, using an ELISA plate reader (SpectraMax 384 Plus, Molecular Devices, USA - Bingol University Central Laboratory Application and Research Center). The cytotoxicity was expressed as the percentage of surviving cells relative to untreated (DMSO) cultures. All experiments were repeated at least three times. The combination index (CI) was calculated in accordance with the Chou-Talalay method [53, 54]. Briefly, CI = (Ares)50/(As)50 + (Bm)50/(Bs)50, where (Ares)50 is the concentration of resveratrol required to induce a 50% inhibitory effect when combined with half of the concentration of the olaparib IC50; (Bres)50 is the IC50 concentration of resveratrol; (Aolap)50 is the concentration of olaparib required to induce a 50% inhibitory effect when combined with half of the concentration of resveratrol IC50; and (Bolap)50 is the IC50 concentration of olaparib. CI values < 1.0 indicates synergy, 1 = additive effect, and > 1.0 antagonism of the combination drug effect [54].

### Crystal violet cell proliferation assay

Crystal violet cell proliferation assay was performed as described [55, 56] with some minor modifications. Briefly, 25 × 10E3 cells were seeded into and treated in 24-well plates and grown for 24 h before treatment. At 8–10 days after seeding, cells were initially fixed in methanol-acetic acid solution (3:1) for 10 min and stained with 0.5% w/v crystal violet solution for 20 min. For the spectrophotometric assay, bound crystal violet was recovered with 1 mL of crystal violet destain solution (10% acetic acid) and the absorbance measured at 595 nm. All experiments were repeated at least three times.

### Neutral red uptake (NRU) assay

The neutral red uptake (NRU) assay was conducted as defined in [57, 58] in order to further examine the in vitro cytotoxic activity of chemical compounds that are investigated. Briefly, cells seeded in a 96-well cell culture plate were treated with the corresponding drugs. At the end of the treatment, the plating medium was aspirated and cells were further incubated with 100 µL of neutral red solution (4 mg/mL stock solution was diluted in plating medium to have 0.4 µg/mL final concentration) added per well for 2 h at 37 °C in a humidified environment with 5% CO_2_. Next, cells were viewed and inspected under an inverted fluorescence microscope (Olympus CKX41) to note the possible precipitation neutral red. Following morphological evaluation of the differences in neutral red uptake, the neutral red solution was aspirated and the remaining solution was washed off by PBS. Next, 150 µL of neutral red destain solution (50% ethanol 96%, 49% deionized water, 1% glacial acetic acid) was added per well and the plate was rapidly shaken using an orbital microplate mixer (Isolab, Germany) for 10 min until the neutral red was recovered from the cells. Readings were measured at 540 nm wavelength in an ELISA plate reader (SpectraMax 384 Plus, Molecular Devices, USA), using blanks which contain no cells as a reference. All experiments were repeated at least three times.

### Colony survival assays for anchorage-dependent growth

Colony survival assays were carried out as reported previously [59]. Briefly, cells were seeded at predetermined densities in 60 mm cell culture plates and allowed to adhere for 24 h, before being drug treated as indicated. At the end of the treatment, cells were washed two times with plating medium. Cells were replenished with fresh complete medium every 3 days until colony size reached more than 50 cells per colony. Cells were then fixed with methanol-acetic acid solution (3:1) for 5 min, followed by staining with 0.5% crystal violet for 15 min. Clusters of ≥ 50 cells were considered as colony. The surviving fraction was calculated using the plating efficiencies of the corresponding non-treated controls as reference [60]. All colony survival experiments were repeated at least three times.

### Soft agar assays for anchorage-independent growth

Soft agar assays were performed as described elsewhere [61] with some modifications. Briefly, 0.6% (w/v) and 1.2% (w/v) ultra-low gelling temperature agaroses were melt in a microwave and cooled to room temperature. Then, equal volumes of the 1.2% melted agarose were mixed with the complete cell culture medium with 20% FBS. After that, 1 mL of 0.6% ultra-low gelling temperature agar was added into each well of the 6-well cell culture plate and set aside to allow agarose to solidify. Cells tripsinised and resuspended in complete medium with 20% FBS (4.000/well) mixed with the equal volume of 0.6% agarose, plated on top of the solidified layer to form colonies in 14–21 days. Cells were replenished with plating medium every 3 days. Colonies stained with 0.05% crystal violet, scanned, and finally quantified using the Image J software. The colony morphology, colony formation capacity and colony size were observed and compared to nontreated samples (DMSO). All colony formation experiments were repeated at least three times.

### Cell migration assays (wound-healing)

In vitro wound-healing assay was performed to determine the cell migration capacity of MCF7 cells upon the corresponding drug treatments as described [62]. Briefly, 5 × 10E5 cells were seeded in 6-well cell culture plates, and after the formation of at least 80% confluent monolayer, a wound was introduced on the surface by scraping with a sterile 200 µL pipette tip. The non-adherent cells were washed off with PBS, and cells were treated with the corresponding drugs for 24 h. At 0, 4, 8, 12 and 24 h after the generation of wounds, treated and non-treated control cells (DMSO) were carefully observed and migration images were obtained with a 10x objective in a phase-contrast inverted microscopy (Motic AE2000). The obtained images were analyzed by Image J software, and wound-closure was calculated by normalizing and comparing 0, 4, 8, 12 and 24 h images of each treatment group with control (DMSO). All experiments were repeated at least three times.

### Immunoblotting and densitometry analysis

Immunoblotting experiments were carried out as in [59]. To detect the relative expression levels of the indicated proteins, samples were resolved by 12% sodium dodecyl sulfate-polyacrylamide gel electrophoresis (SDS- PAGE) and transferred to polyvinylidene difluoride membranes (Immobilon^®^-P PVDF Membrane, Millipore, #IPVH00010). Following protein transfer, membranes were blocked with TBST (50 mM Tris, 150 mM NaCl, 0.5% Tween 20, at pH 7.5) containing 5% skim milk powder and then incubated overnight with the corresponding antibody. Bound antibodies were detected by horseradish peroxidase-conjugated secondary antibodies. Membranes were eventually processed with an enhanced chemiluminescence reagent (ECL solution in an equal mix of solution A (2.5 mM luminol (Sigma-Aldrich, #123072), 39.6 mM coumaric acid (Sigma-Aldrich, #C9008), 100 mM Tris-HCl, pH 8.5) and solution B (100 mM Tris-HCl, pH 8.5 and 0.062% H2O2), and the x-ray film (Fujifilm Medical X-ray Film Blue Sensitive Super RX-N, #47410) images were developed in a dark room. Densitometric analysis of immunoblots were performed using the ImageJ software (NIH).

### DNA ladder assay

The DNA fragmentation assay procedure for evaluating cell death by detecting DNA fragments using agarose gel electrophoresis was performed as reported [62]. Briefly, genomic DNA (gDNA) isolation was carried out according to the manufacturer’s instructions (Hibrigen Genomic DNA Isolation Kit, #MG-DHDNA). The concentration of gDNA was determined in a spectrophotometer (BioDrop, Cambridge, UK - Bingol University Bee and Natural Products R&D and P&D Application and Research Center). Finally, 30 µg of each extracted DNA sample was loaded onto a 1% agarose gel (Serva, #11404.03) containing RedSafe™ DNA staining solution (Intronbio, #21141) and the experiment carried out by a horizontal electrophoresis system (Owl™ EasyCast™ B2 Mini Gel Electrophoresis Systems, Thermo Scientific, USA). All agarose DNA gel electrophoresis was visualized and analyzed by BioRad Molecular Imager Gel Doc XR+ (BioRad, Hercules, CA).

### Acridine orange/ethidium bromide (AO/EB) staining

Acridine orange/ethidium bromide (AO/EB) staining was performed as instructed [63] in order to visualize nuclear fragmentation of apoptotic cells. 25 µL of cell suspension (5 × 10E5 cells/mL) was mixed with 1 µL of AO/EB solution prior to microscopic analysis. 10 µL of cell-stain suspension was transferred onto a microscopic slide, which was then covered with a glass coverslip. Finally, control and drug-treated cells were viewed and examined under an inverted fluorescence microscope (Olympus CKX41). At least 300 cells were counted to quantify apoptosis.

### Theoretical methods

Theoretical computations provide significant insights into the chemical and biological characteristics of molecules. A multitude of quantum chemical parameters is derived via theoretical computations. The computed parameters elucidate the chemical behaviors of the molecules. A variety of programs are used to compute molecular structures. The programs are Gaussian09 RevD.01 and GaussView 6.0 [64, 65]. Calculations were conducted using the B3LYP, HF, and M06-2x techniques with the 6–31 g basis established by these programs. Consequently, several quantum chemical parameters have been identified by these simulations. Each parameter delineates a distinct chemical feature of molecules; the computed parameters are derived as follows [66]:

$$\:\chi\:=-{\left(\frac{\partial\:{{\rm\:E}}}{\partial\:{{\rm\:N}}}\right)}_{\upsilon\:\left(r\right)}=\frac{1}{2}\left(I+A\right)\cong\:\frac{1}{2}({E}_{HOMO}+{E}_{LUMO})$$

$$\:\eta\:=-{\left(\frac{{\partial\:}^{2}{{\rm\:E}}}{\partial\:{{{\rm\:N}}}^{2}}\right)}_{\upsilon\:\left(r\right)}=\frac{1}{2}\left(I-A\right)\cong\:-\frac{1}{2}\left({E}_{HOMO}-{E}_{LUMO}\right)$$

$$\:\sigma\:=1/\eta\:\:\:\:\:\:\:\:\:\:\omega\:={\chi\:}^{2}/2\eta\:\:\:\:\:\:\:\:\:\:\:\:\:\:\:\epsilon\:=1/\omega\:$$

Molecular docking simulations are conducted to evaluate the biological activities of compounds in relation to biological substrates. The Maestro Molecular Modeling Platform (version 13.4) created by Schrödinger [67] was used for molecular docking calculations. Calculations consist of several stages. Each step is executed individually. The protein preparation module [68, 69] was used in the protein preparation process. This module identified the active sites of the proteins. The subsequent phase involves the preparation of the examined compounds. Initially, the molecules are optimized using the Gaussian software program, after which the LigPrep module [70] is configured for calculations using the optimized structures. The Glide ligand docking module [71, 72] was used to investigate the interactions between the compounds and the cancer protein after preparation. All computations were conducted with the OPLS4 technique. Ultimately, ADME/T study (absorption, distribution, metabolism, excretion, and toxicity) will be conducted to evaluate the pharmacological potential of the investigated compounds. The Qik-prop module of the Schrödinger program [73] was used to forecast the impacts and interactions of chemicals inside human metabolism.

### Statistical analysis

Graphics and statistical analyses were carried out using the Graph-Pad Prism software. Data are presented as mean ± S.E.M., unless stated otherwise. Statistical significance was assessed applying one-tailed un-paired Student’s t-test unless stated otherwise. For all tests, differences were considered statistically significant when p-values were below 0.05 (*), 0.01 (**), or 0.001 (***), respectively.

## Results

### Resveratrol augments in vitro cytotoxic activity of Olaparib in breast cancer cells

**Fig. 1:**
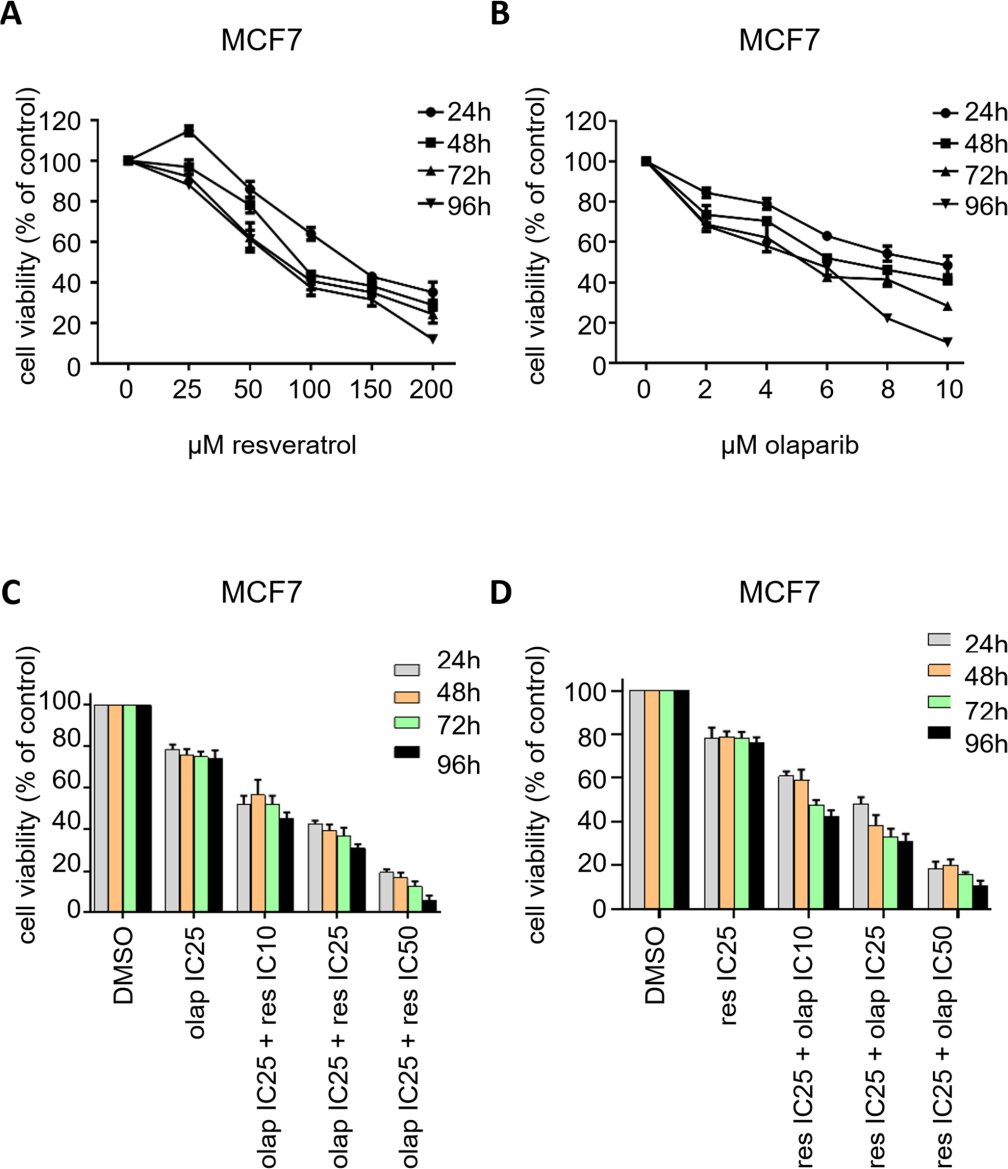
WST-1 cell viability assays show that resveratrol and olaparib combination synergistically decrease the viability of MCF7 breast cancer cells

As a colorimetric test, WST-1 (2-(4-iodophenyl)−3-(4-nitrophenyl)−5-(2,4-disulfo- phenyl)−2 H-tetrazolium, monosodium salt) assay is one of the fundamental in vitro cytotoxicity techniques and it is extensively used in drug screening studies to assess the cytotoxic potential of anti-neoplastic agents [74]. In our research, we performed WST-1 assays to investigate the synergistic cytotoxicity of resveratrol and olaparib combination treatment in MCF7 breast cancer cells, used as a BRCA wild-type cell line model in our cytotoxicity investigation [75, 76]. We initially assessed the individual cytotoxic effects of different concentrations of resveratrol and olaparib in MCF7 cells. Cells seeded in 96-well plates were cultured overnight and treated with increasing doses of resveratrol or olaparib for 24 h, 48 h, 72 h and 96 h. There was a time and dose dependent reduction in the MCF7 cell viability with resveratrol or olaparib alone treatments (Fig. 1A-B). The IC50 concentrations of resveratrol were calculated as follows: 124.55 ± 4.23; 98.98 ± 4.37; 82.45 ± 1.50 and 76.86 ± 2.76 µM for 24 h, 48 h, 72 h and 96 h timepoints, respectively. The IC50 concentrations of olaparib were calculated as follows: 9.60 ± 0.68; 7.08 ± 0.31; 5.24 ± 0.23 ve 4.85 ± 0.26 µM for 24 h, 48 h, 72 h and 96 h timepoints, respectively. As noted, higher concentrations of resveratrol resulted in a significant reduction in the viability of MCF7 cells. Interestingly, the combination of resveratrol and olaparib caused a significant decrease in the viability of cells compared to the alone treatments (Fig. 1C-D). Based on these data, the combination index of 24 h, 48 h, 72 h and 96 h treatments were calculated as 1.31; 0.91; 0.67 ve 0.62, revealing a synergistic interaction between resveratrol and olaparib for 48 h, 72 h and 96 h treatment periods. We decided to use 72 h timepoint for all of the following experiments.

**Fig. 2:**
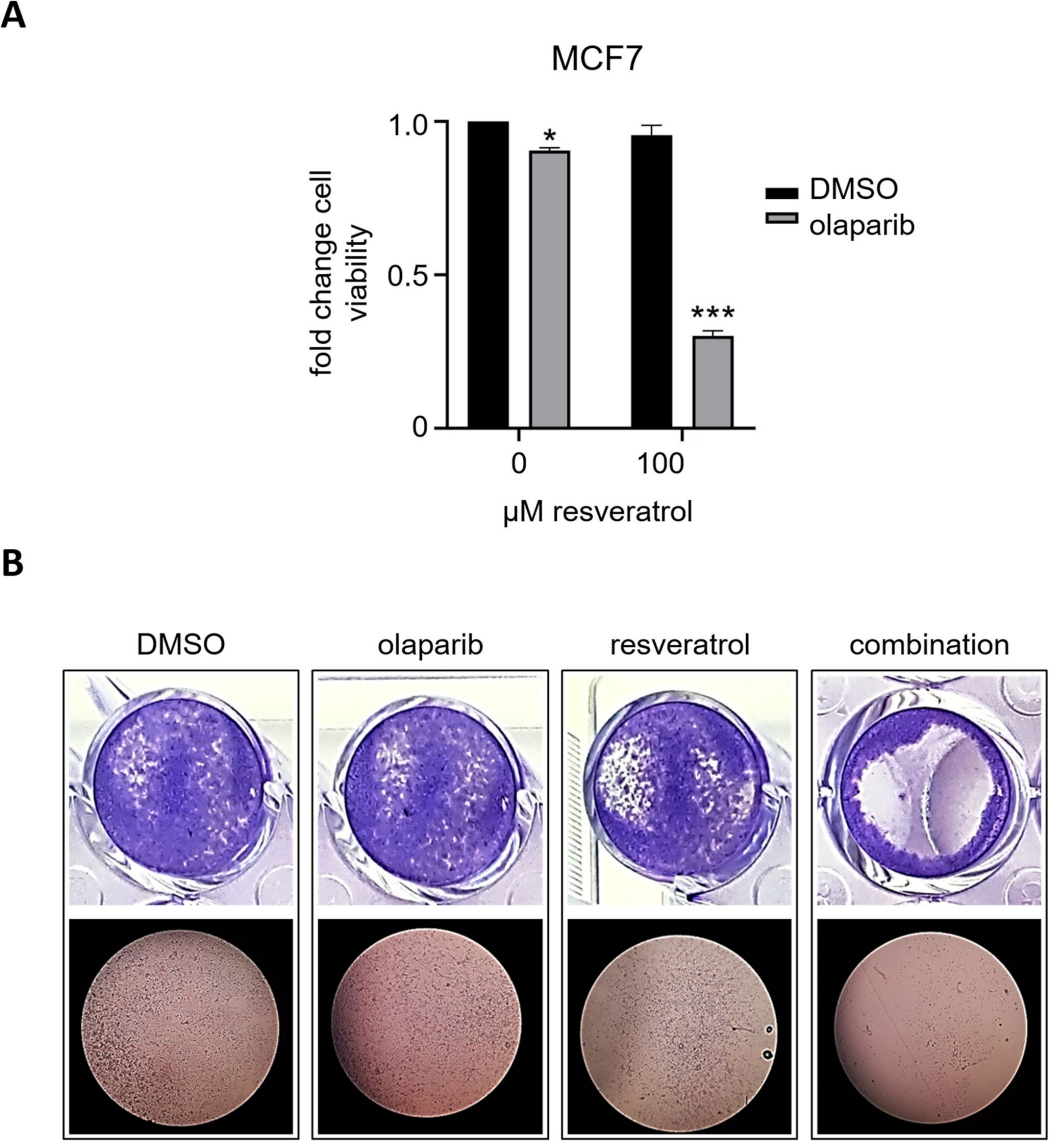
Crystal violet cell viability assays show that resveratrol and olaparib combination decrease the viability of MCF7 breast cancer cells

We next conducted in vitro crystal violet cell proliferation assays in order to validate the synergism in the resveratrol and olaparib combination. The assay is based on washing off the detached dead cells and staining live cells attached to plates [55, 56]. MCF7 cells were seeded in 24-well plates and the following day treated with/out resveratrol (100 µM) in absence or presence of olaparib (1.25 µM) for 72 h. As seen in Fig. 2A-B, olaparib treatment slightly reduced the MCF7 cell viability, whereas the cytotoxic effect was significantly potentiated when olaparib combined with resveratrol, signifying a synergistic effect of the olaparib and resveratrol combination.

**Fig. 3:**
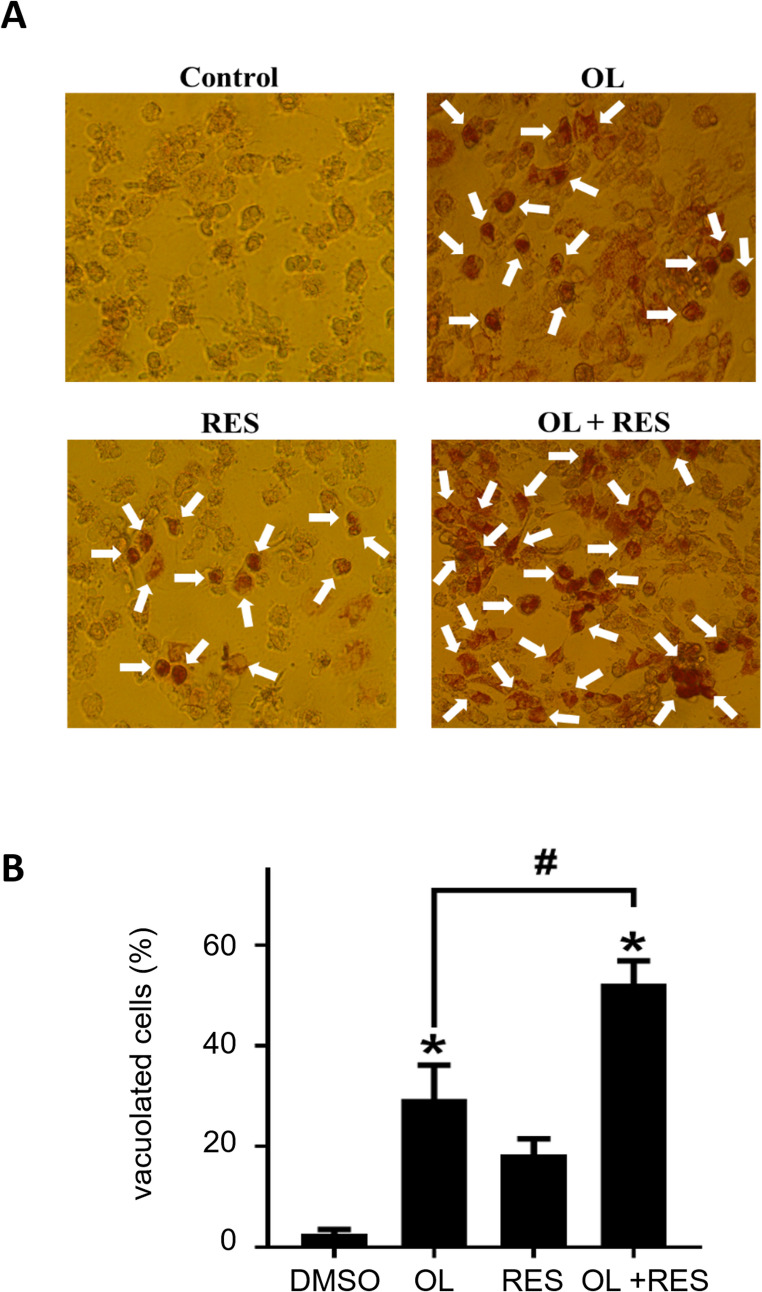
Neutral red staining of olaparib (OL), resveratrol (RES) and olaparib + resveratrol (OL + RES) treated MCF7 cells. The images were obtained by using a inverted microscope. (**A**) MCF7 cells were treated 5 µM olaparib, 50 µM resveratrol, and 5 µM olaparib + 50 µM resveratrol for 72 h. Then the cells were stained with neutral red. There were no vacuoles in untreated cells (control), while vacuole formation was observed in the treatment groups. The acidic vacuoles, which associated with apoptosis following autophagy, in cells were red. White arrows indicate vacuolated cells. (**B**) The percentages of vacuolated cells formed were calculated and compared with the control. Experiments were performed in triplicate and standard deviation values were calculated and the results were given as mean ± standard deviation (SD). Statistical evaluation of the findings obtained as a result of the studies was made using the GraphPad Prism version 8.00 (GraphPad Software, San Diego California USA) program. Findings were evaluated with t-test and One-Way Anova test. Results with a P-value less than 0.05 (*) (#) were considered as significant

To consolidate our previous results, we finally performed neutral red uptake assay. The assay is one of the most used cytotoxicity tests in drug screening studies and offers a quantitative assessment of the number of viable cells in a cell culture [57, 58]. Cells seeded in 96-well plates were treated with the corresponding drugs for 72 h and the assay was processed as described. As expected, compared to individual treatments, there was a synergically significant cytotoxic effect in the combination of resveratrol and olaparib (Fig. 3A-B). Taken together, in vitro cell viability and proliferation assays we conducted reveals a potential combinational synergism between resveratrol and olaparib treatments in MCF7 breast cancer cells.

### Resveratrol combined with olaparib inhibits 2D clonogenic survival and 3D tumorigenic formation of breast cancer cells

**Fig. 4:**
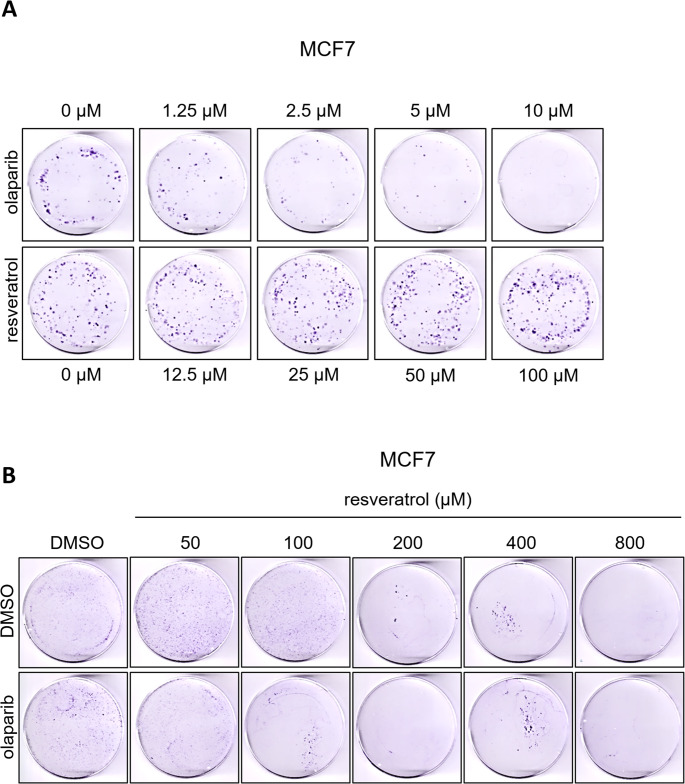
Colony survival assays reveal that resveratrol and olaparib combination reduce the survival of MCF7 breast cancer cells

**Fig. 5:**
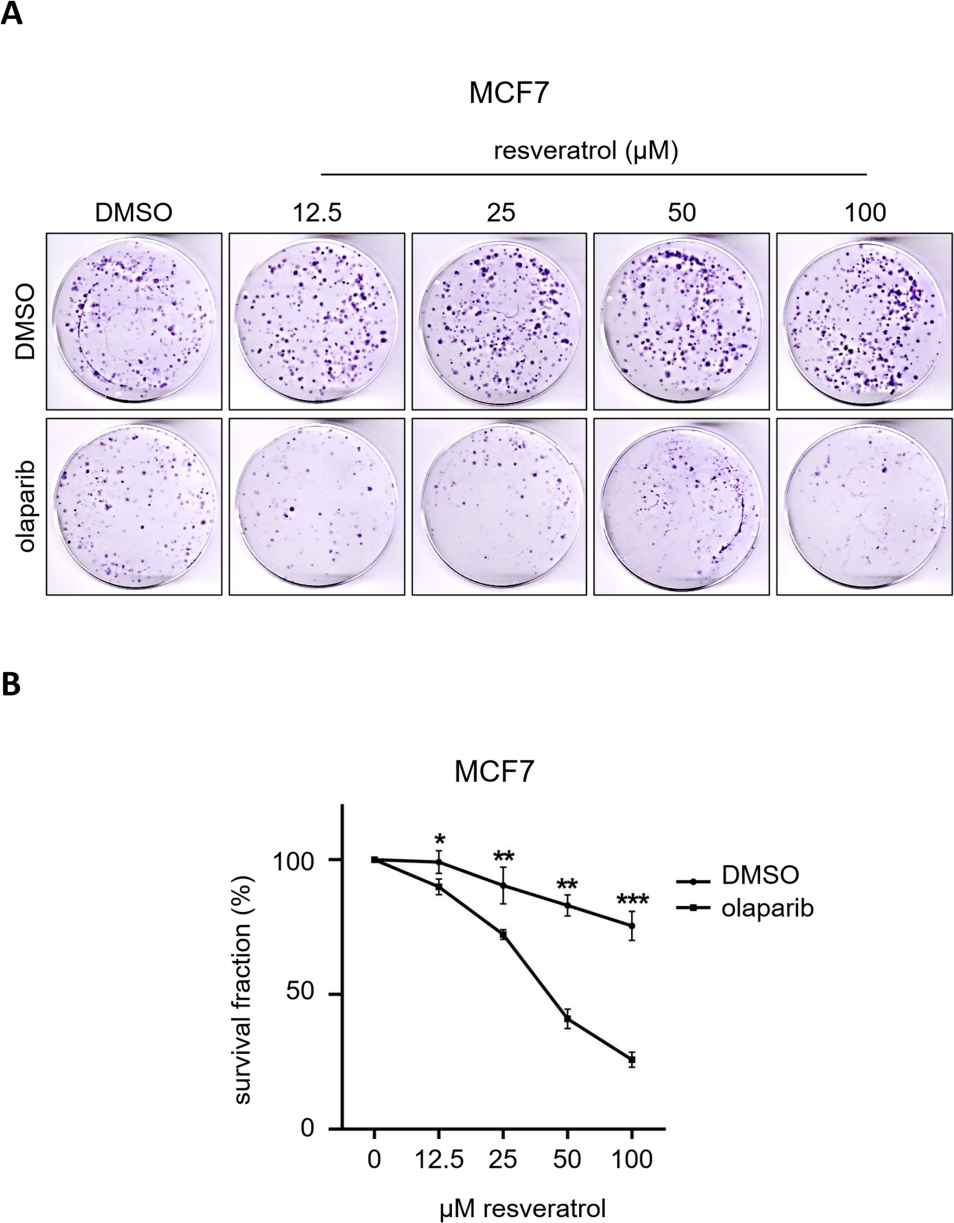
Colony survival assays reveal that resveratrol and olaparib combination reduce the survival of MCF7 breast cancer cells. Results with a P-value less than 0.05 (*), 0.01 (**) or 0.001 (***) were considered as significant

We performed in vitro anchorage-dependent growth assays in order to evaluate the long-term effects of the resveratrol and olaparib combination on the clonogenic survival of MCF7 breast cancer cells. The assay aims to determine and compare the fraction of cultured cancer cells which maintain the prolonged capacity to produce colonies in the absence or presence of therapeutic agents [60]. Cells seeded in 60 mm dishes were treated with the corresponding drugs 72 h and incubated further for colony formations. Our initial individual treatment tests showed that the clonogenic survival of MCF7 cells significantly reduced in olaparib dose dependent manner, whereas no effect was noted in all concentrations of resveratrol up to 100 µM (Fig. 4A). Then we decided to use 1.25 µM concentration of olaparib combined with increasing doses of resveratrol up to 800 µM and, in line with other reports [19, 20], observed that very high concentrations of resveratrol (≥ 200 µM) tremendously cytotoxic to MCF7 cancer cells (Fig. 4B). Since the lower concentrations of resveratrol was enough to potentiate the cytotoxic activity of olaparib (Fig. 4B), we eventually treated cells with 12.5, 25, 50 and 100 µM resveratrol with/out olaparib (1.25 µM) for 72 h and observed that the multiple concentrations of resveratrol effectively intensified the effects of olaparib (Fig. 5A-B).

**Fig. 6:**
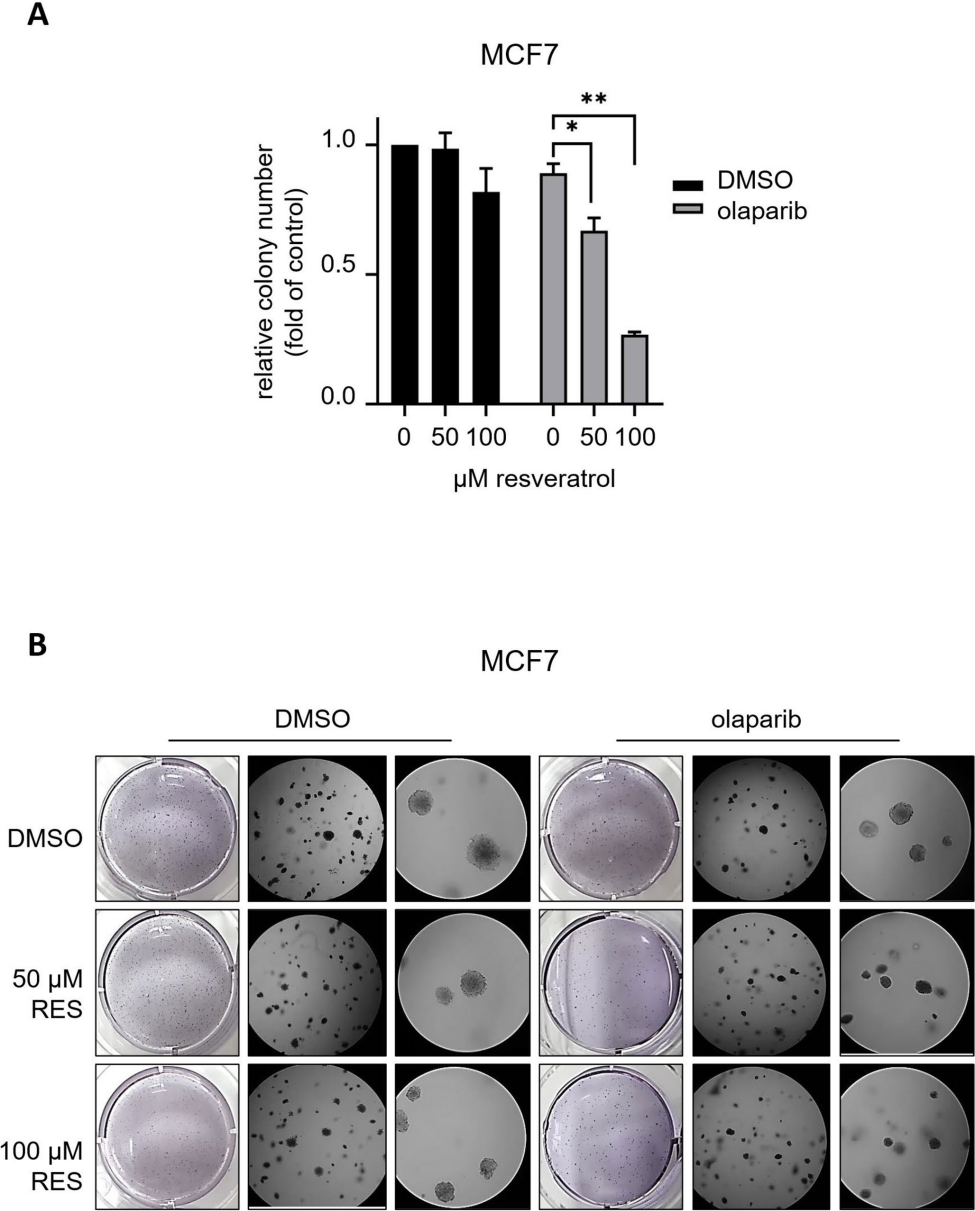
Anchorage-independent tumour formation assays reveal that resveratrol and olaparib combination reduce the survival of MCF7 breast cancer cells. Results with a P-value less than 0.05 (*) or 0.01 (**) were considered as significant

Since anchorage-independent colony formation is a characteristic feature of neoplastic cells which best correlates with tumorigenesis [77, 78], we next conducted in vitro tumour growth assays in soft agar in order to assess the long-term effects of the resveratrol and olaparib combination on the anchorage-independent growth of MCF7 breast cancer cells. Following treatment with indicated doses of resveratrol (0–50–100 µM) with/out olaparib (1.25 µM) for 72 h, cells trypsinised and diluted in soft agar were plated in 6-well plates and regularly supplied with fresh medium containing the relevant drugs. As expected, resveratrol did not interfere with the tumour formation ability of MCF7 cells (Fig. 6A-B). Although the individual olaparib treatment slightly shrunk the tumour sizes (qualitative observation), there was no significant decrease in the number of colonies formed (Fig. 6A-B). However, compared to olaparib alone treatment, we observed a statistically significant reduction in the number of colonies formed when cells were subjected to resveratrol treatment (50–100 µM) in combination with olaparib (1.25 µM) (Fig. 6A-B). Indeed, the cytotoxic effect was more striking when 100 µM resveratrol was combined with 1.25 µM olaparib, and the colony formation was reduced by approximately 3-fold compared to olaparib treatment alone (Fig. 6A-B). Consistent with the number of colonies, the combination of increasing doses of resveratrol with olaparib effectively shrunk the size of tumours formed during 3–4 weeks (qualitative observation). Consequently, our in vitro clonogenic survival and tumorigenic formation results further confirms that resveratrol potentiates the cytotoxicity of olaparib in breast cancer cells, suggesting a potential synergism in the combination therapy.

### Resveratrol and olaparib combination reduce migration of MCF7 cancer cells

**Fig. 7:**
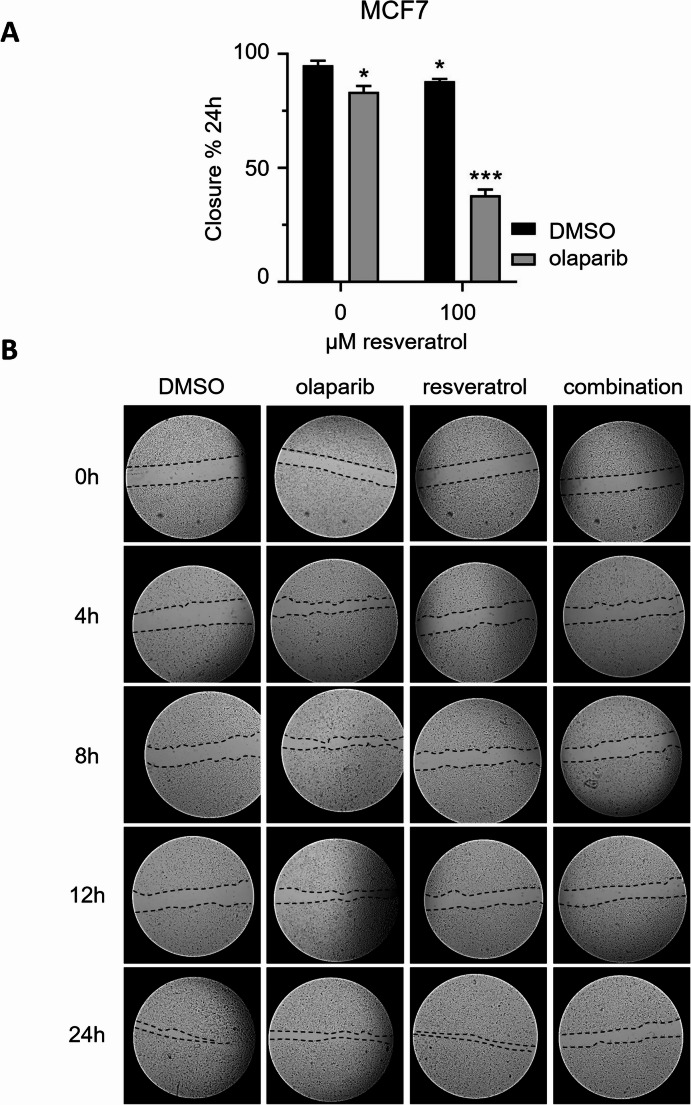
Wound-healing assay demonstrates that resveratrol and olaparib combination reduce the migration of MCF7 breast cancer cells. Results with a P-value less than 0.05 (*) or 0.001 (***) were considered as significant

Cell migration is a critical process involved in normal physiology including embryonic development, angiogenesis and wound repair, and in several pathological conditions including inflammation, and cancer invasion and metastasis [79, 80]. Since the anti-migration potentials of therapeutic agents and combination therapies are correlated with their anti-cancer capacity [80], we performed wound-healing assays, a highly effective and reproducible technique to study cancer cell migration in vitro. Considering the previous cytotoxicity results, we decided to use the most effective combination concentration of drugs in our wound-healing assays: 100 µM and 1.25 µM for resveratrol and olaparib, respectively. MCF7 cancer cells were seeded at a high density in 6-well plates and in the following day, a scratch wound was created on the cell surface using a sterile micropipette tip. The corresponding cells were treated with the indicated concentrations of drugs for 24 h and the migration images at certain timepoints after the wound creation were obtained with an inverted microscopy. As displayed in Fig. 7A, monotherapy with 100 µM resveratrol or 1.25 µM olaparib brought about statistically significant decreases in the percentage of wound closure at 24 h, whereas the combination of resveratrol and olaparib was more effective, and the rate of wound-closure was attenuated by approximately 2-fold compared to individual treatments (Fig. 7A-B). In summary, our results suggest that resveratrol strengthens the anti-cancer activities of PARP inhibitor olaparib.

### Resveratrol and olaparib combination induces DNA damage and activates apoptosis

**Fig. 8:**
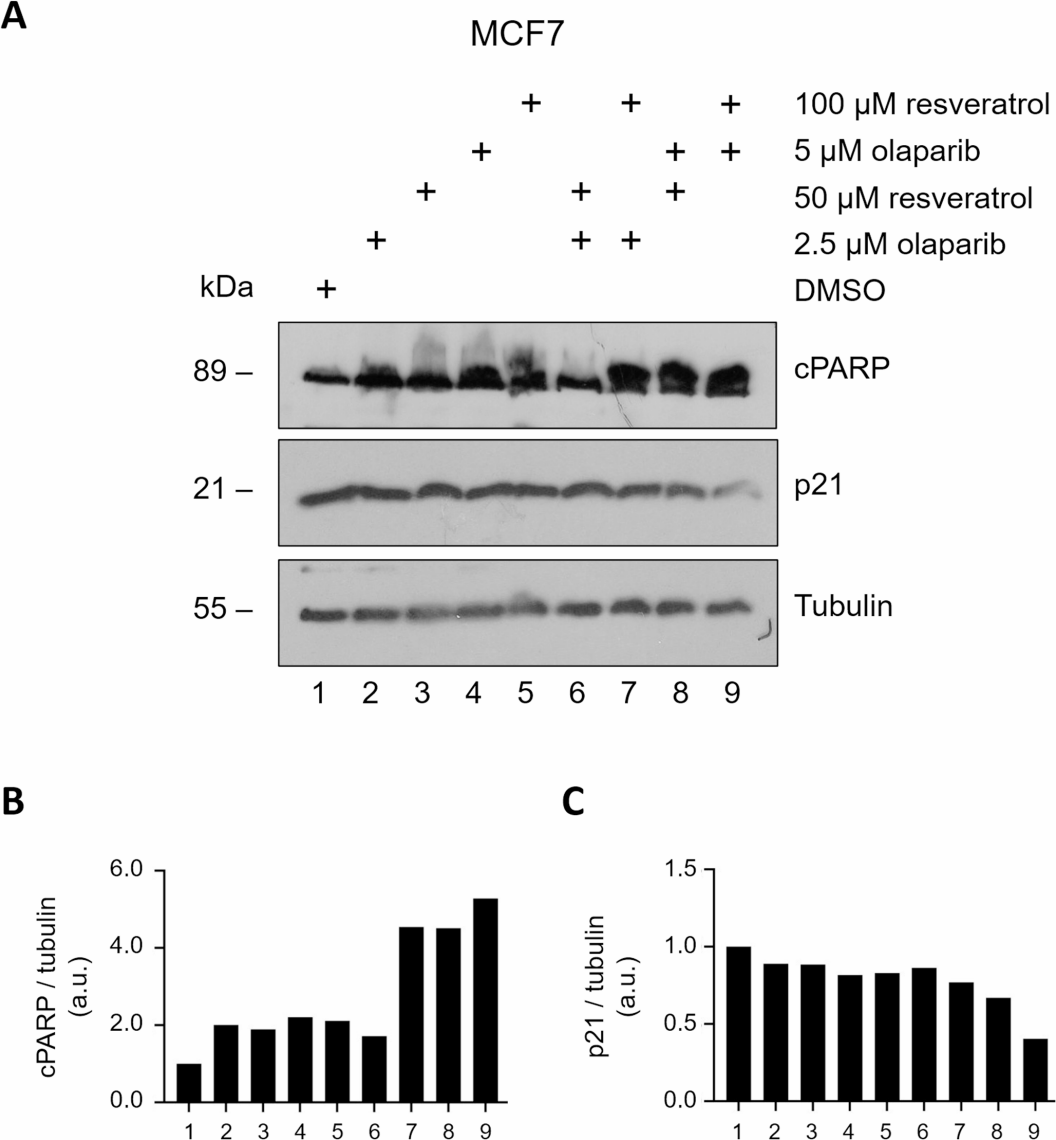
Resveratrol and olaparib combination induce apoptosis in MCF7 breast cancer cells

To understand the cellular mechanisms underlying our biological findings obtained so far, we analysed the post-translational expression profiles of the well-known regulatory proteins involved in cell cycle and apoptotic cell death processes via immunoblotting technique. The PARP enzyme is one of the key target proteins subjected to a specific caspase cleavage, resulting in the formation of 24 kDa and 89 kDa fragments, during apoptosis. Hence, the 89 kDa cleaved PARP (cPARP) fragments serve as apoptotic biomarker [81, 82]. The expression of a key cell cycle regulator, p21^WAF1/CIP1^, is mainly controlled by the p53 tumour suppressor protein, therefore, p21 is responsible for p53-dependent cell cycle arrest through CDK inhibition following DNA damage induction [83]. In our immunoblotting experiments, we employed a cPARP-specific antibody to detect the apoptotic activation, and a p21-specific antibody to evaluate the status of cell cycle regulation. MCF7 cells seeded in 100 mm dishes were next day treated with various concentration of resveratrol (0–50–100 µM) and olaparib (0–2.5–5 µM) alone or in combination for 24 h. After total protein purification, samples were subjected to 12% SDS-PAGE separation and consequent Western blot analysis using the respective antibodies. Compared to untreated control groups, all drug treatments resulted in a significant increase in the amount of cPARP, however, higher concentrations of the combined drug treatments more intensely augmented the extent of cPARP formation, with an estimated 4-fold increase (Fig. 8A, B; line 7, 8, 9). Interestingly, the combination of 2.5 µM olaparib and 50 µM resveratrol did not produce the same amount of cPARP (an almost one-fold increase) compared to other combination treatments (Fig. 8A, line 6), which could be explained by the limited sensitivity of the immunoblotting method. Remarkably, the p21 protein expression was strikingly downregulated in cells treated with the co-treatment of resveratrol and olaparib (Fig. 8A, C).

**Fig. 9:**
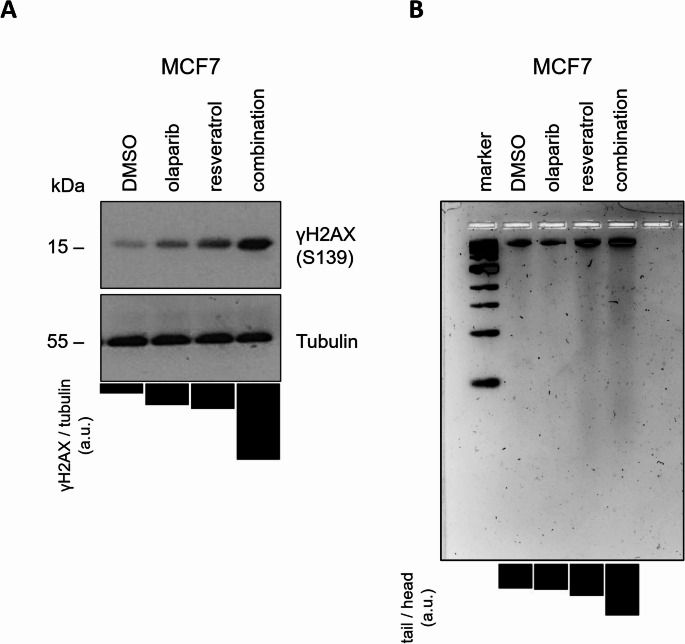
Resveratrol and olaparib combination causes DNA damage and apoptosis in MCF7 breast cancer cells

As a FDA-approved and clinically used PARP inhibitor, olaparib inhibits the repair of DNA SSBs, and therefore, indirectly cause the accumulation of lethal DSBs especially occurred during replication. Although HR-proficient cancer cells can repair the respective DSBs, HR-deficient cancer cells cannot handle the overwhelming burden of accumulated DSBs and subjected to death [84–86]. Recent studies revealed that resveratrol could inhibit the expression of key HR proteins and render cancer cells vulnerable to various anti-cancer agents [19, 20, 22]. Based on these data, we tried to understand why resveratrol and olaparib combination produces synergistic cytotoxicity through apoptosis. We hypothesized that resveratrol may attenuate the efficacy of HR, which is required to repair detrimental DSBs induced by olaparib treatment. To probe whether apoptosis was occurred upon resveratrol and olaparib combination as a consequence of elevated DNA damage levels, we examined the expression of γH2AX (Ser139) which serves as a biomarker for the accumulation of DSBs [87]. As shown in Fig. 9A, the levels of phosphorylated γH2AX (Ser139) was increased in response to resveratrol (100 µM) or olaparib (2.5 µM) treatments for 24 h and the upregulation was more severe when the combination of resveratrol (100 µM) and olaparib (2.5 µM) treatment was applied for 24 h.

The fragmentation of the large genomic DNA into smaller oligomers is one of the classic features of apoptosis and visualizing these DNA fragments is judged as an apoptotic event [62]. Hence, we carried out DNA ladder (fragmentation) assays to confirm whether the resveratrol and olaparib combination induced apoptotic cell death in MCF7 cells. Cells seeded in 60 mm plates were treated with resveratrol (100 µM) and/or olaparib (2.5 µM) for 24 h. Following day, total genomic DNA was isolated and subjected to agarose gel electrophoresis, revealing more visible smear pattern in comparison to untreated and individual treatments (Fig. 9B).

**Fig. 10:**
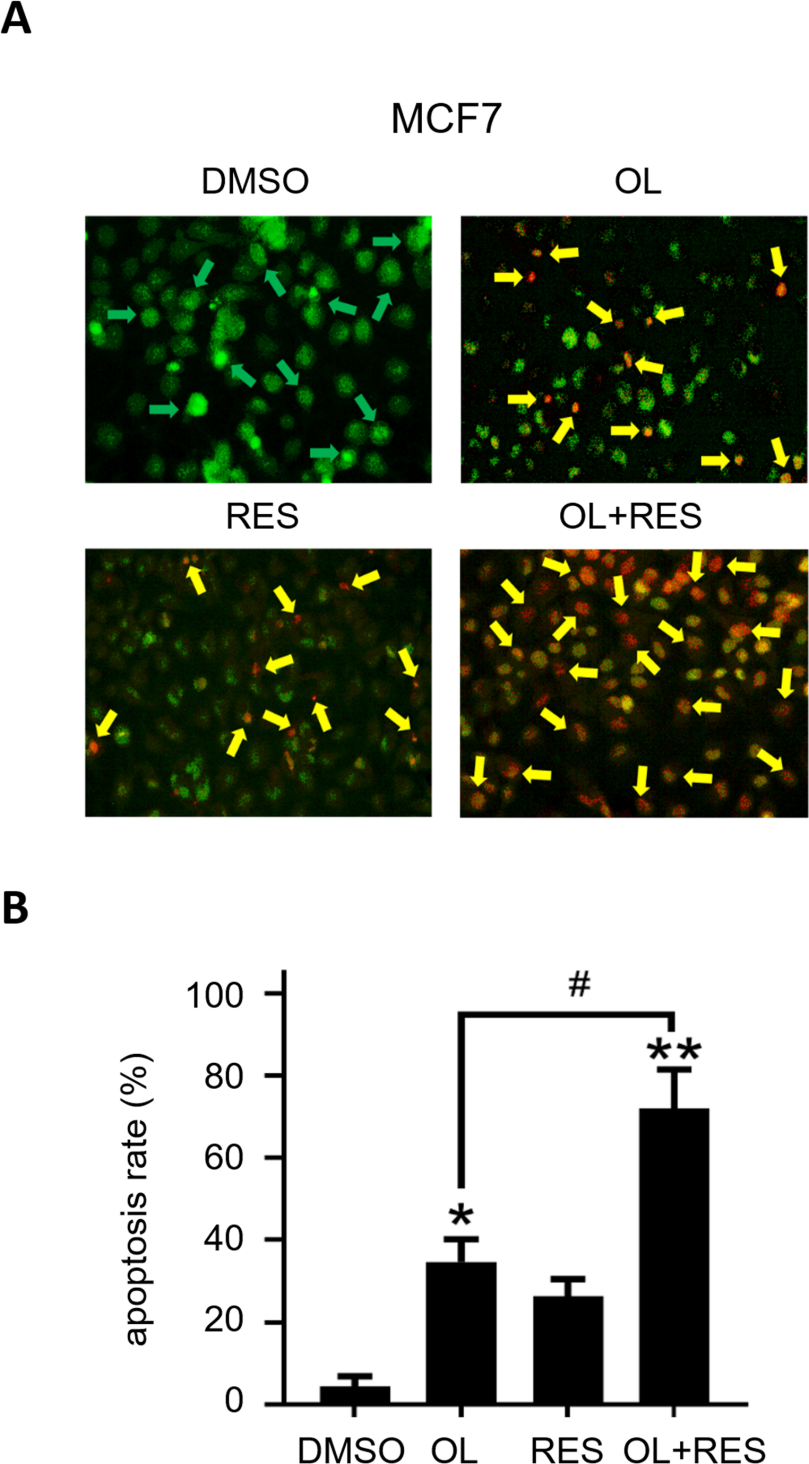
Morphological observation of MCF7 breast cancer cells treated with olaparib (OL), resveratrol (RES) and olaparib + resveratrol (OL + RES) after AO/EB double staining. **(A)** 1 × 10^4^ MCF7 cells were seeded in 96-well microplates and the next day cells were treated for 72 h with untreated (control), 5 µM olaparib, 50 µM resveratrol, and 5 µM olaparib + 50 µM resveratrol. The treatment was then terminated and cells were stained with AO/EB solution. The morphological change in the cells was visualized under a fluorescence-attached inverted microscope (Olympus CKX41). Green arrows indicate viable cells, yellow arrows indicate apoptotic cells. **(B)** Percentages of apoptotic cells formed as a result of the applied treatments were calculated. Experiments were performed in triplicate and standard deviation values were calculated and the results were given as mean ± standard deviation (SD). Statistical evaluation of the findings obtained as a result of the studies was made using the GraphPad Prism version 8.00 (GraphPad Software, San Diego California USA) program. Findings were evaluated with t-test and One-Way Anova test. Results with a P-value less than 0.05 (*) (#) or 0.01 (**) were considered as significant

Last but not least, we performed acridine orange/ethidium bromide (AO/EB) labelling assay, which is routinely employed to visualize nuclear fragmentation of apoptotic cells as a characteristic of apoptosis [63]. MCF7 cells seeded in 60 mm dishes were treated with resveratrol (100 µM) and/or olaparib (2.5 µM) for 24 h. Next day, cells were mixed with AO/EB solution and analysed under an inverted fluorescence microscope. Our findings showed that there was a significant increase in the fraction of apoptotic cells in the combination group compared to untreated and individual treatment groups (Fig. 10A-B). Overall, our results suggest that the combination of resveratrol and olaparib may lead to synergistic cytotoxicity by inducing apoptosis in breast cancer cells.

### Theoretical calculations

knowledge the electronic structure and chemical characteristics of molecules requires a knowledge of concepts like the ΔE energy gap, E_HOMO_, E_LUMO_, chemical hardness, softness, electronegativity, and chemical potential. These are the fundamental metrics used to evaluate a chemical’s reactivity and stability.

As seen in reference [88], the energy gap, represented by the symbol ΔE, measures the difference in energy levels between a molecule’s highest occupied molecular orbital (HOMO) and lowest unoccupied molecular orbital (LUMO). A thorough comprehension of this value is a vital condition for comprehending a molecule’s electrical stability and reactivity. Higher molecular reactivity is indicated by a small ΔE value, while a larger ΔE value suggests a more stable molecular structure [89].

The acronyms E_HOMO_ and E_LUMO_ stand for the energy of the highest occupied molecular orbital and the lowest unoccupied molecular orbital, respectively, in the context of molecular orbital energy. The HOMO controls a molecule’s capacity to give electrons, or nucleophilic behavior, whereas the LUMO controls a molecule’s ability to take electrons, or electrophilic behavior [90]. A thorough comprehension of the energy difference between the HOMO and LUMO states is necessary to comprehend the mechanics of electron transport in chemical reactions.

Chemical hardness is the ability of a molecule to withstand change brought on by outside influences. More stable structures and less reactivity are characteristics of molecules with higher hardness. A measure of a molecule’s reactivity is its chemical softness; soft molecules are more vulnerable to changes in their chemical composition. Hardness and softness notions may be used to get an understanding of the acid-base characteristics of molecules and the ability to forecast the development of chemical bonds.

A key factor in determining the polarity of chemical bonding is electronegativity. It is a term used to describe an atom’s or molecule’s propensity to draw bonding electrons. During chemical processes, molecules with high electronegativity are better at attracting electrons and displaying electrophilic properties.

Chemical potential [91], as described in, shows how the system reacts to variations in electron density and quantifies the energy shift inside a molecule. When analyzing reaction energy and molecule stability, this number is an essential signal.

**Fig. 11:**
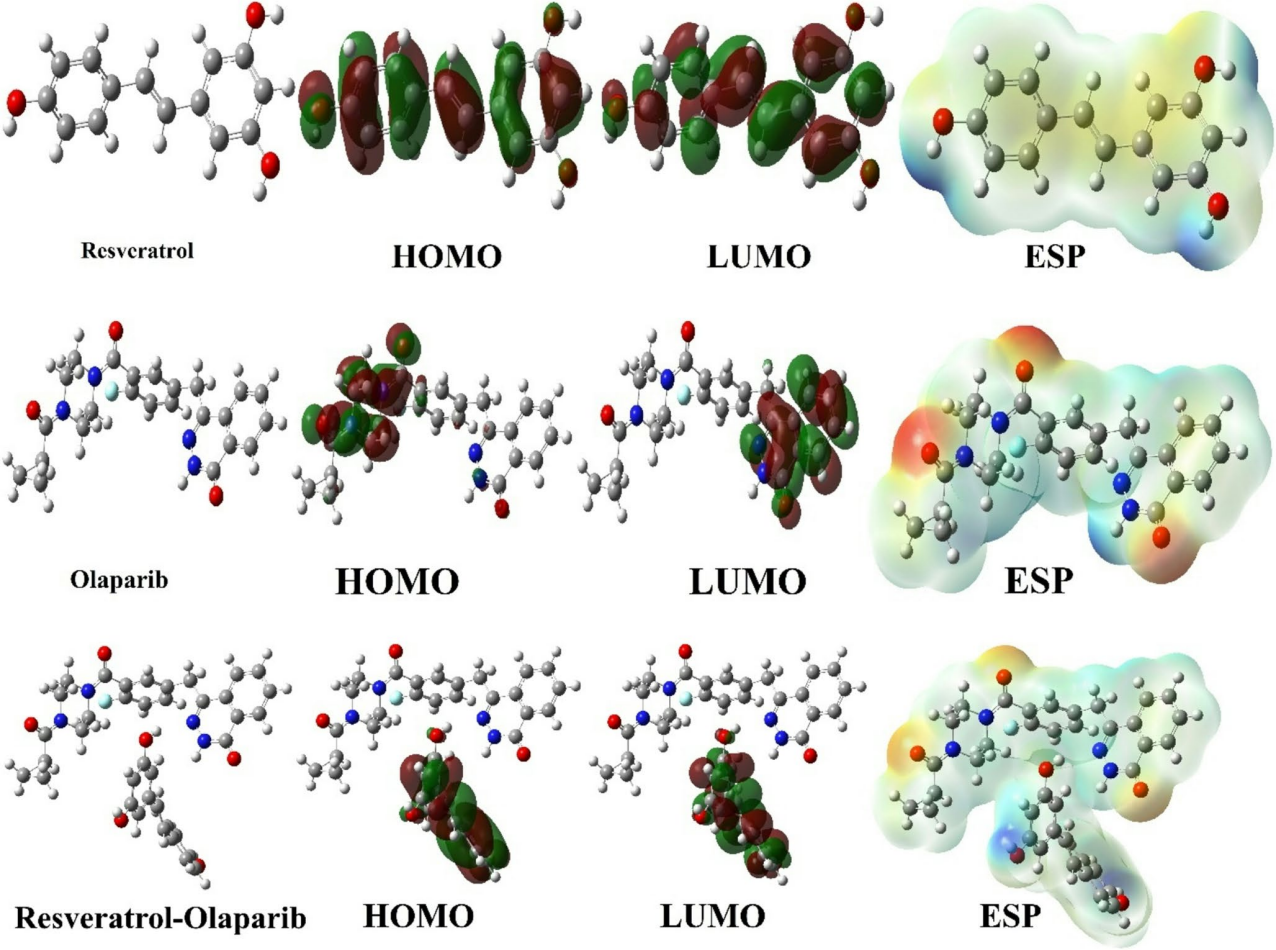
Representations of optimize structure, HOMO, LUMO, and ESP of molecules

When combined, these terms provide a thorough foundation for comprehending the stability, chemical reactivity, and electrical structure of molecules. When combined with ΔE, the evaluation of the HOMO and LUMO states’ energy levels provides a very useful technique for predicting molecular reactivity [92]. In order to understand how molecules respond to external stimuli, properties such as electronegativity, softness, and chemical hardness are essential. Table 1; Fig. 11 offer a detailed list of all the parameters.

**Table 1:** The calculated quantum chemical parameters of molecules

	E_HOMO_		E_LUMO_		I		A		ΔE		η		µ		χ		PA		ω		ε		dipol		Energy
B3LYP/6–31 g LEVEL																									
R	−5.3748		−1.3189		5.3748		1.3189		4.0559		2.0279		0.4931		3.3469		−3.3469		2.7618		0.3621		3.010		−20843.4383
O	−6.2685		−1.7399		6.2685		1.7399		4.5286		2.2643		0.4416		4.0042		−4.0042		3.5405		0.2824		5.614		−40058.9584
R-O	−5.4927		−1.9304		5.4927		1.9304		3.5623		1.7811		0.5614		3.7115		−3.7115		3.8670		0.2586		7.287		−60902.3491
B3LYP/6–31 + + g LEVEL																									
R	−5.6984		−1.7184		5.6984		1.7184		3.9800		1.9900		0.5025		3.7084		−3.7084		3.4554		0.2894		3.086		−20844.4542
O	−6.6062		−2.1211		6.6062		2.1211		4.4850		2.2425		0.4459		4.3637		−4.3637		4.2456		0.2355		6.228		−40060.6080
R-O	−5.8306		−2.2466		5.8306		2.2466		3.5840		1.7920		0.5580		4.0386		−4.0386		4.5508		0.2197		7.508		−60904.9049
B3LYP/6–31 + + g(d, p) LEVEL																									
R	−5.5852		−1.6357		5.5852		1.6357		3.9495		1.9747		0.5064		3.6104		−3.6104		3.3005		0.3030		2.667		−20850.7228
O	−6.6481		−1.9916		6.6481		1.9916		4.6564		2.3282		0.4295		4.3198		−4.3198		4.0076		0.2495		5.353		−40072.8083
R-O	−5.7071		−2.2556		5.7071		2.2556		3.4515		1.7258		0.5795		3.9813		−3.9813		4.5925		0.2177		7.932		−60923.3086
HF/6–31 g LEVEL																									
R		−7.6544		2.2822		7.6544		−2.2822		9.9366		4.9683		0.2013		2.6861		−2.6861		0.7261		1.3772		2.931	
O		−9.2416		1.8893		9.2416		−1.8893		11.1309		5.5655		0.1797		3.6762		−3.6762		1.2141		0.8237		6.819	
R-O		−7.7534		1.8047		7.7534		−1.8047		9.5581		4.7790		0.2092		2.9744		−2.9744		0.9256		1.0804		7.803	
HF/6–31 + + g LEVEL																									
R		−7.8133		0.9086		7.8133		−0.9086		8.7219		4.3609		0.2293		3.4523		−3.4523		1.3665		0.7318		2.948	
O		−9.4141		0.7448		9.4141		−0.7448		10.1589		5.0795		0.1969		4.3347		−4.3347		1.8495		0.5407		7.043	
R-O		−7.9725		0.6985		7.9725		−0.6985		8.6710		4.3355		0.2307		3.6370		−3.6370		1.5255		0.6555		7.921	
HF/6–31 + + g(d, p) LEVEL																									
R		−7.6761		0.9508		7.6761		−0.9508		8.6269		4.3134		0.2318		3.3627		−3.3627		1.3107		0.7629		2.491	
O		−9.2726		0.8008		9.2726		−0.8008		10.0735		5.0367		0.1985		4.2359		−4.2359		1.7812		0.5614		5.766	
R-O		−7.8100		0.7244		7.8100		−0.7244		8.5344		4.2672		0.2343		3.5428		−3.5428		1.4707		0.6799		6.894	
M062X/6–31 g LEVEL																									
R		−6.7131		−0.4158		6.7131		0.4158		6.2973		3.1487		0.3176		3.5644		−3.5644		2.0176		0.4956		2.971	
O		−7.7357		−0.7793		7.7357		0.7793		6.9564		3.4782		0.2875		4.2575		−4.2575		2.6057		0.3838		5.662	
R-O		−6.7917		−1.4136		6.7917		1.4136		5.3781		2.6891		0.3719		4.1027		−4.1027		3.1297		0.3195		10.671	
M062X/6–31 + + g LEVEL																									
R		−6.9594		−0.3129		6.9594		0.3129		6.6464		3.3232		0.3009		3.6362		−3.6362		1.9893		0.5027		3.019	
O		−7.9782		−1.1222		7.9782		1.1222		6.8560		3.4280		0.2917		4.5502		−4.5502		3.0199		0.3311		6.185	
R-O		−6.8628		−1.6569		6.8628		1.6569		5.2059		2.6029		0.3842		4.2598		−4.2598		3.4857		0.2869		11.537	
M062X/6–31 + + g(d, p) LEVEL																									
R		−6.8584		−0.7197		6.8584		0.7197		6.1387		3.0693		0.3258		3.7891		−3.7891		2.3388		0.4276		2.560	
O		−7.8927		−0.9992		7.8927		0.9992		6.8935		3.4468		0.2901		4.4460		−4.4460		2.8674		0.3487		5.046	
R-O		−6.7781		−1.4681		6.7781		1.4681		5.3101		2.6550		0.3766		4.1231		−4.1231		3.2015		0.3124		10.116	

R: Resveratrol, O: Olaparib, R-O: Resveratrol and Olaparib combination

According to Koopman’s theorem [93], a key idea in molecular orbital theory, a molecule’s ionization energy and electron affinity are related to the energy levels of its HOMO (highest occupied molecular orbital) and LUMO (lowest unoccupied molecular orbital). The idea is that the HOMO’s energy is the molecule’s ionization energy, and the LUMO’s energy is a reflection of the molecule’s electron affinity. This method is an effective way to accurately forecast the electrical characteristics and reactivity of molecules. However, since the theory ignores electron-electron interactions, the findings are just an approximation. The results could need more intricate computations.

The Hard and Soft Acid-Base concept (HSAB) [94] was created to improve understanding of acid-base chemistry. According to this theory, strong interactions happen when hard acids and hard bases come into contact, whereas strong interactions happen when soft acids and soft bases use hard acids. On the other hand, “hard” refers to characteristics like high ionization energy, small size, and low polarizability, whereas “soft” refers to characteristics like low polarizability, big size, and high polarizability.

According to the Maximum Hardness Principle (MHP) [95], chemical systems favor forming structures with the highest possible hardness. According to this theory, a system will often take on a structure that is more rigid. Because of its increased hardness, the improved structure is more stable and less reactive. This concept serves as a fundamental foundation for forecasting the stability of molecules and the reactions that take place during chemical reactions. The creation of reactive intermediates and the assessment of transition states often make use of the maximal hardness concept.

These three fundamental ideas provide both theoretical and practical underpinnings for a comprehensive knowledge of molecular reactivity and bonding properties. Mechanisms behind electron transport are examined using Koopman’s theorem. The properties of acid-base interactions are explained using the HSAB paradigm. Additionally, PMH plays a major role in molecular stability prediction [94]. Combining these methods allows for a thorough comprehension of the behavior of chemical systems.

Quantum chemical parameters of the molecule series consisting of resveratrol, olaparib, and resveratrol and olaparib combinations were calculated. As a result of the calculations, when the molecules were compared according to the HOMO energy numerical values, it was seen that the Resveratrol molecule had higher activity than the other molecules at all levels. When the molecules were compared according to the LUMO energy numerical values, it was seen that the Resveratrol and olaparib combination had higher activity than the other molecules at all levels. When the molecules were ranked according to the ∆E energy gap numerical value, it was seen that the Resveratrol and olaparib combination had higher activity than the other molecules at all levels. Finally, when the molecules were compared according to the electronegativity numerical value, it was seen that the Resveratrol molecule had higher activity than the other molecules at all levels.

In Table 1, each molecule has unique energy levels and numerical values for a range of properties. These compounds were combined to create new and more potent molecular groups, but Table 2’s thermodynamic characteristics showed no indications of spontaneous synthesis. Table 3 also included the numerical values for every molecule. The following equations were used to calculate ∆E, ∆H, and ∆G:

$$\:\varDelta\:E=\sum\:{E}_{complex}-\sum\:{E}_{molecule}$$

$$\:\varDelta\:H=\sum\:{E}_{complex}-\sum\:{E}_{molecule}$$

$$\:\varDelta\:G=\sum\:{E}_{complex}-\sum\:{E}_{molecule}$$

**Table 2:** Numerical value of the molecules’ physicochemical parameters

		B3LYP			
		*R*	O	*R*-O	
E	6–31 g	−20841.8448	−40056.5352	−60898.8669	
	6–31 + + g	−20842.8521	−40058.1673	−60901.3875	
	6–31 + + g (d, p)	−20849.1162	−40070.3196	−60919.6442	
H	6–31 g	−20841.8191	−40056.5095	−60898.8412	
	6–31 + + g	−20842.8264	−40058.1416	−60901.3618	
	6–31 + + g (d, p)	−20849.0905	−40070.2939	−60919.6185	
G	6–31 g	−20843.4383	−40058.9584	−60902.3491	
	6–31 + + g	−20844.4542	−40060.6080	−60904.9049	
	6–31 + + g (d, p)	−20850.7228	−40072.8083	−60923.3086	
		**HF**			
		**R**	**O**	**R-O**	
E	6–31 g	−20841.8448	−40056.5352	−60898.8669	
	6–31 + + g	−20713.0699	−39810.1080	−60523.5077	
	6–31 + + g (d, p)	−20721.8494	−39827.8287	−60549.8331	
H	6–31 g	−20712.4083	−39808.9912	−60521.7511	
	6–31 + + g	−20713.0442	−39810.0823	−60523.4820	
	6–31 + + g (d, p)	−20721.8237	−39827.8030	−60549.8074	
G	6–31 g	−20713.9609	−39811.3459	−60525.1499	
	6–31 + + g	−20714.6065	−39812.4503	−60526.9219	
	6–31 + + g (d, p)	−20723.3931	−39830.1997	−60553.3576	
		**M062X**			
		**R**	**O**	**R-O**	
E	6–31 g	−20841.8448	−40056.5352	−60898.8669	
	6–31 + + g	−20834.2375	−40042.2324	−60877.4472	
	6–31 + + g (d, p)	−20840.1622	−40053.8488	−60894.7898	
H	6–31 g	−20833.4115	−40040.8758	−60875.1150	
	6–31 + + g	−20834.2118	−40042.2067	−60877.4215	
	6–31 + + g (d, p)	−20840.1365	−40053.8231	−60894.7642	
G	6–31 g	−20835.0021	−40043.3102	−60878.4310	
	6–31 + + g	−20835.8113	−40044.6472	−60880.6970	
	6–31 + + g (d, p)	−20841.7476	−40056.2826	−60898.1050

**Table 3:** Numerical value of the molecules’ physicochemical parameters

		B3LYP	HF	M062X
∆G	6–31 g	0.0476	0.1568	−0.1187
	6–31 + + g	0.1574	0.1349	−0.2385
	6–31 + + g(d, p)	0.2225	0.2352	−0.0748
∆H	6–31 g	−0.5126	−0.3517	−0.8277
	6–31 + + g	−0.3939	−0.3555	−1.0030
	6–31 + + g(d, p)	−0.2340	−0.1807	−0.8046
∆E	6–31 g	−0.4869	−0.3260	−0.8020
	6–31 + + g	−0.3682	−0.3298	−0.9773
	6–31 + + g(d, p)	−0.2083	−0.1550	−0.7789

A key thermodynamic parameter, energy E represents the sum of E0, Evib, Erot, and Etransl energies and includes both electronic and thermal energies. On the other hand, Energy H stands for the total of all energy types existing inside the building. These two prior total energy values are subtracted to get the energy G [96].

In terms of ΔG (Gibbs free energy change), positive values are seen in some method and basis set combinations and negative values in others. Negative ΔG indicates that the process tends to be spontaneous, while positive ΔG indicates that it is thermodynamically non-spontaneous in Table 4 [97]. The fact that ΔH (enthalpy change) values are mostly negative indicates that the process is exothermic. The electronic energy change (ΔE) results are also mostly negative; HF and M06X often predict more negative values than B3LYP, suggesting that this system is more stable [98].

**Table 4:** Numerical values of the Docking parameters of molecules against proteins

1A52	Resveratrol	Olaparib	Resveratrol and Olaparib combination
Docking Score	−8.10	−5.33	-
Glide ligand efficiency	−0.48	−0.17	-
Glide hbond	−0.88	−0.52	-
Glide evdw	−23.07	−44.31	-
Glide ecoul	−9.17	−2.50	-
Glide emodel	−39.22	−56.01	-
Glide energy	−32.25	−46.81	-
Glide einternal	2.08	8.34	-
Glide posenum	119	313	-
1JNX	**Resveratrol**	**Olaparib**	**Resveratrol and Olaparib combination**
Docking Score	−4.28	−3.69	−4.22
Glide ligand efficiency	−0.25	−0.12	−0.09
Glide hbond	−0.27	−0.16	−0.57
Glide evdw	−14.51	−31.20	−33.22
Glide ecoul	−11.16	−5.61	−12.76
Glide emodel	−33.84	−44.96	−55.11
Glide energy	−25.67	−36.80	−45.97
Glide einternal	0.68	3.05	5.68
Glide posenum	304	291	363

As a result, the calculated energy values and thermodynamic parameters become more reliable with the expansion of the basis set (e.g. 6–31 + G(d) or 6–311 + + G(d, p)). The differences between the methods arise from the fact that each method treats electron correlation and long-range interactions differently. Therefore, it is necessary to compare with experimental data or more advanced calculations to determine which method and basis set gives the closest results to reality.

**Fig. 12:**
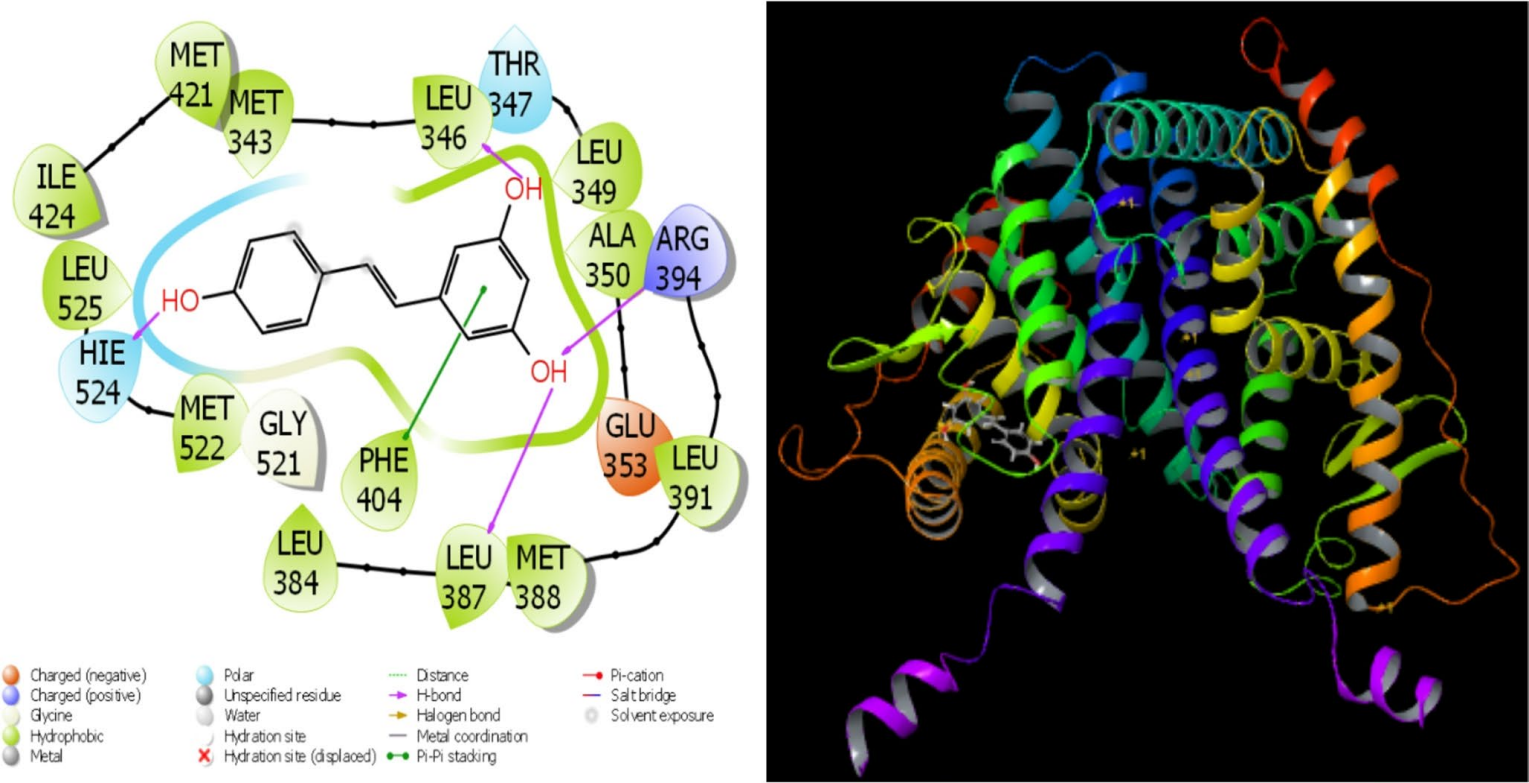
Presentation interactions of Resveratrol with 1A52 protein

**Fig. 13:**
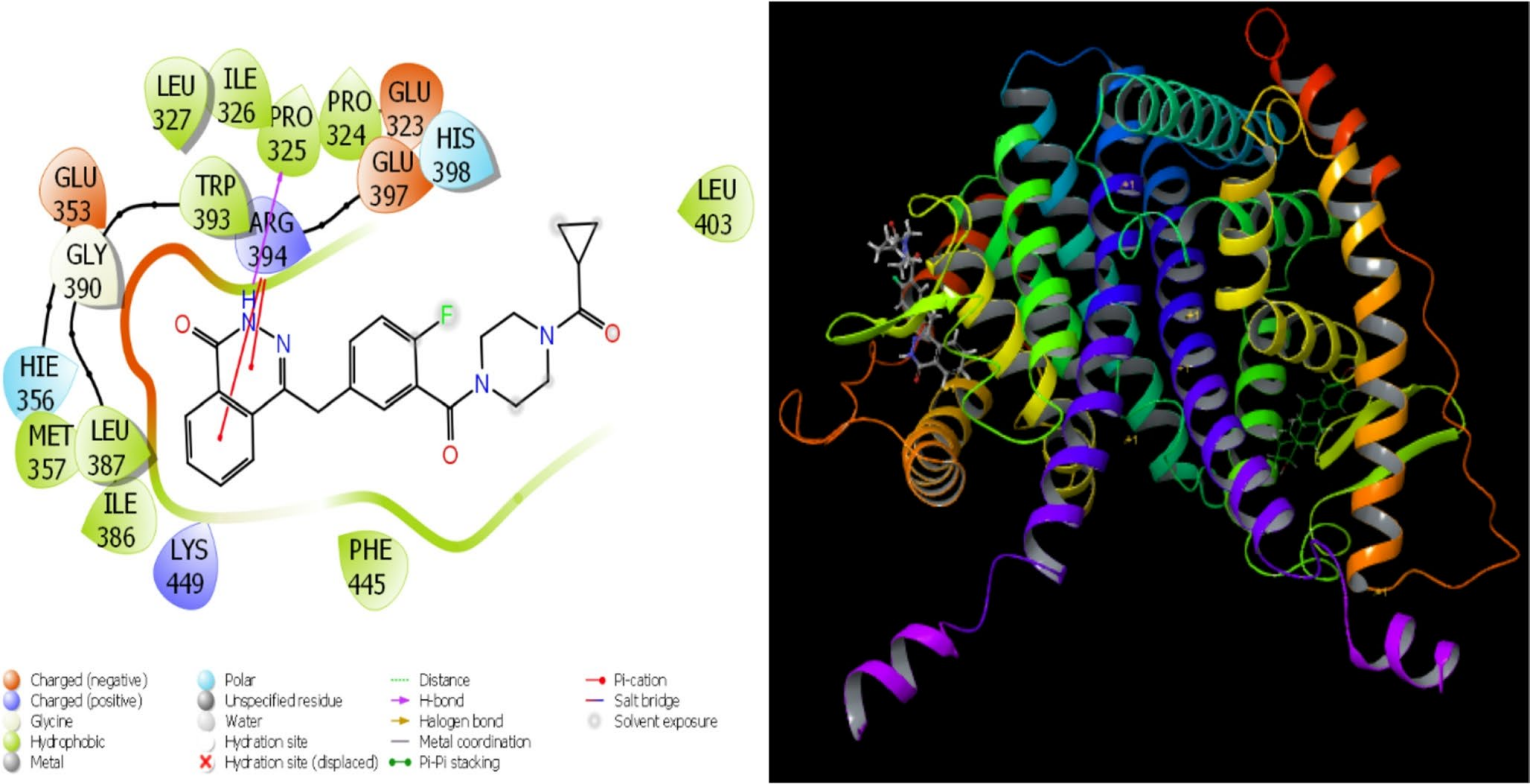
Presentation interactions of Olaparib with 1A52 protein

**Fig. 14:**
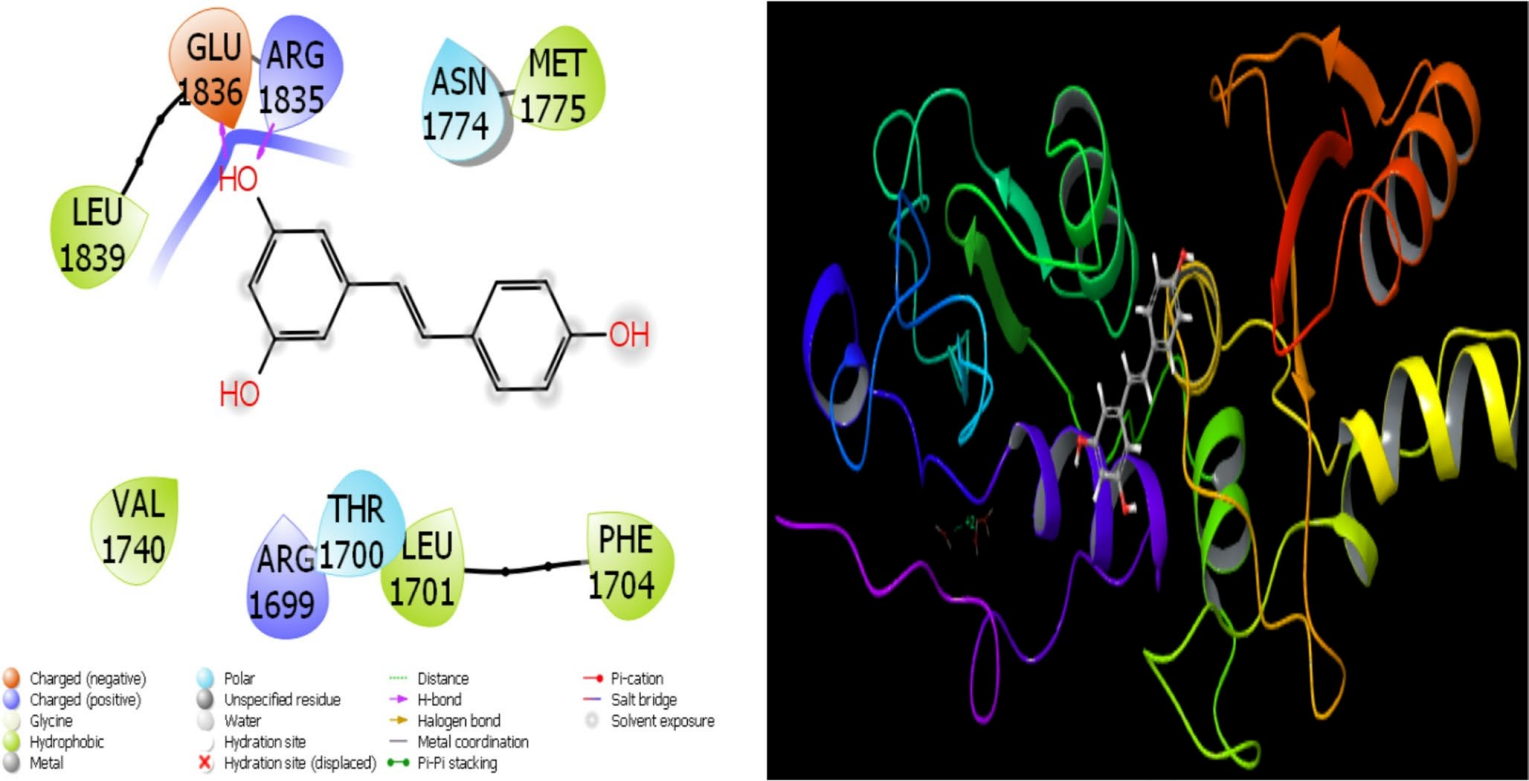
Presentation interactions of Resveratrol with 1JNX protein

**Fig. 15:**
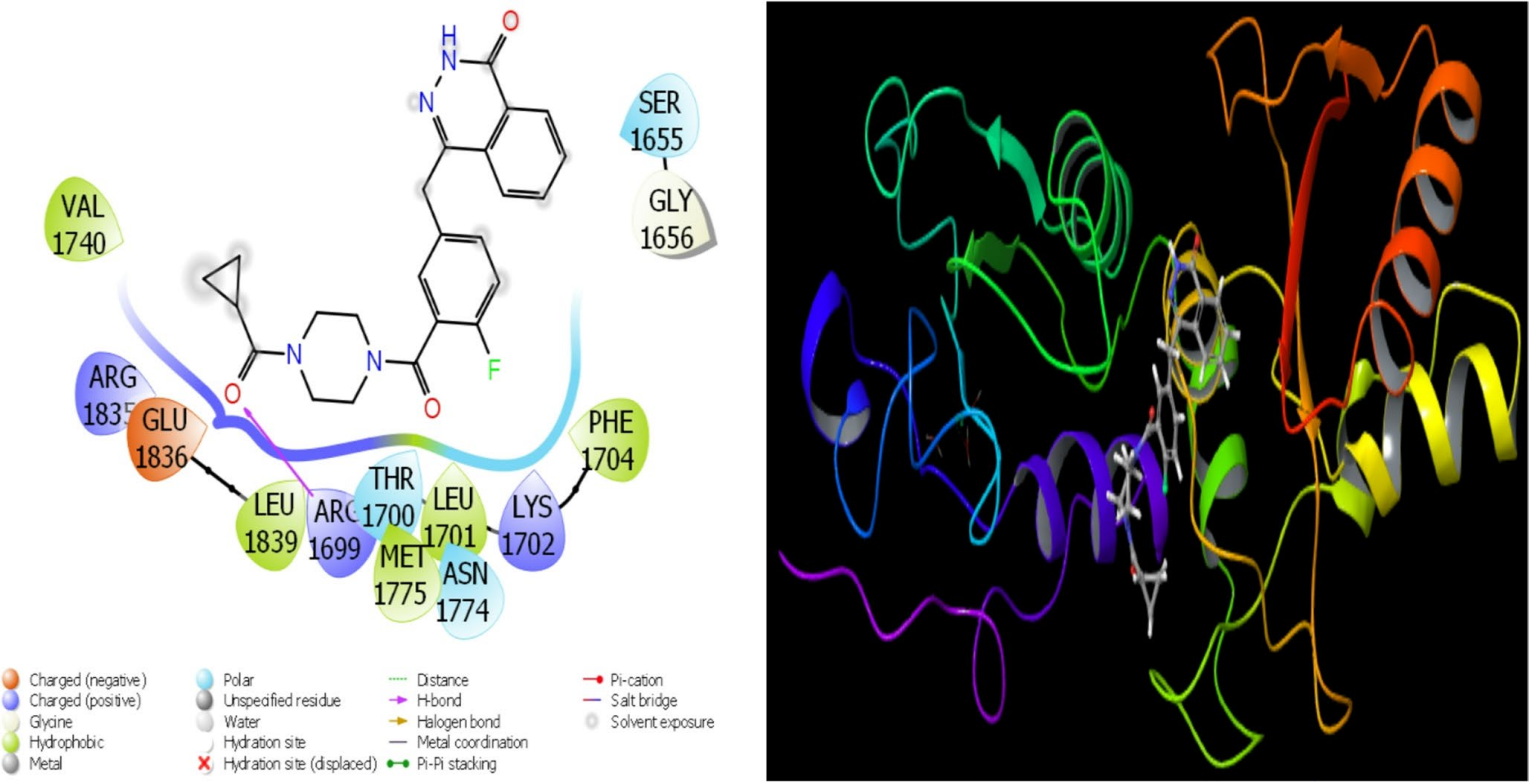
Presentation interactions of Olaparib with 1JNX protein

**Fig. 16:**
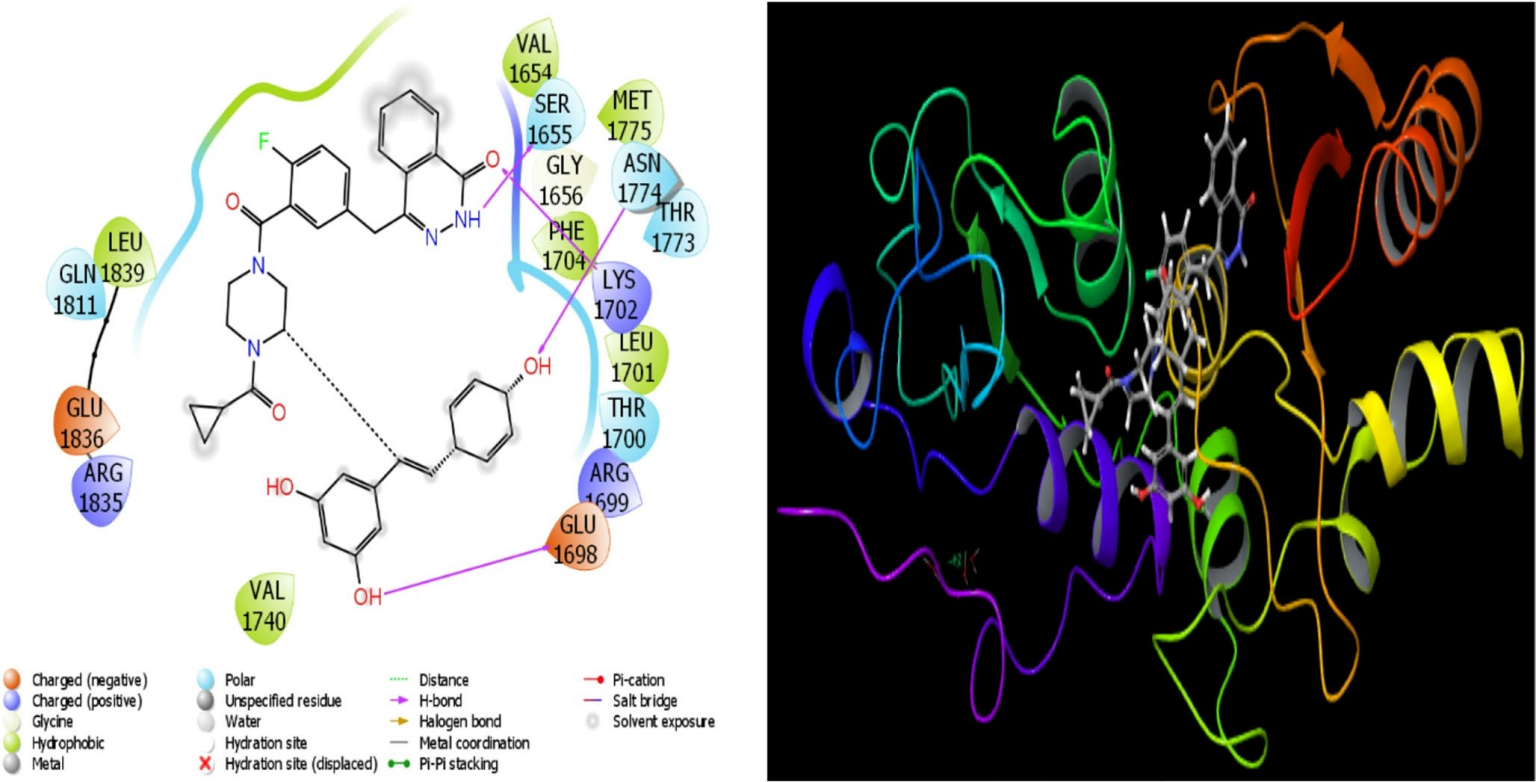
Presentation interactions of Resveratrol and Olaparib combination with 1JNX protein

According to recent studies, the broad use of theoretical research and technological improvements has sped up and simplified the comparison of molecular biological processes [99]. The use of computations has greatly accelerated and improved the process of identifying the most successful and effective medications before experimental testing. Certain parameters were identified as a consequence of the theoretical calculations. Using quantitative values of many factors, this method assesses the biological activity of substances. The interactions between certain proteins and substances are the main factor influencing the activities in question. These interactions are so pervasive that they eventually prevent the proteins from working as intended. This is the process via which inhibition takes place. The interactions between proteins and other molecules define the energy levels of molecules. Hydrogen bonds, polar and hydrophobic contacts, π-π interactions, and halogen interactions are the ways that molecules and proteins interact [100, 101]. The preservation of equilibrium depends on molecular interactions. A thorough analysis of these chemical interactions shows that there are several ways in which molecules and proteins interact with one another. All parameters are included in Table 5, while all examples are shown in Figs. 12, 13, 14, 15 and 16.

**Table 5:** ADME properties of molecules

	Resveratrol	Olaparib	Resveratrol and Olaparib combination	Referance Range
mol_MW	228	434	-	130–725
dipole (D)	2.7	7.4	-	1.0–12.5.0.5
SASA	480	724	-	300–1000
FOSA	18	277	-	0–750
FISA	163	156	-	7–330
PISA	298	256	-	0–450
WPSA	0	35	-	0–175
volume (A^3^)	781	1323	-	500–2000
donorHB	3	1	-	0–6
accptHB	2	8	-	2.0–20.0
glob (Sphere = 1)	0.9	0.8	-	0.75–0.95
QPpolrz (A^3^)	24.1	46.7	-	13.0–70.0
QPlogPC16	9.3	13.4	-	4.0–18.0
QPlogPoct	13.5	22.1	-	8.0–35.0
QPlogPw	9.3	14.9	-	4.0–45.0
QPlogPo/w	2.0	2.9	-	−2.0-6.5
QPlogS	−2.8	−5.0	-	−6.5-0.5
CIQPlogS	−3.4	−5.1	-	−6.5-0.5
QPlogHERG	−5.3	−4.5	-	*
QPPCaco (nm/sec)	280	193	-	**
QPlogBB	−1.3	−1.2	-	−3.0-1.2
QPPMDCK (nm/sec)	125	231	-	**
QPlogKp	−2.9	−3.1	-	Kp in cm/hr
IP (ev)	8.6	9.3	-	7.9–10.5
EA (eV)	0.6	1.0	-	−0.9-1.7
#metab	3	1	-	1–8
QPlogKhsa	−0.2	0.1	-	−1.5-1.5
Human Oral Absorption	3	3	-	-
Percent Human Oral Absorption	82	85	-	***
PSA	67	110	-	7–200
RuleOfFive	0	0	-	Maximum is 4
RuleOfThree	0	0	-	Maximum is 3
Jm	0.5	0.0	-	**-**

* corcern below − 5, **<25 is poor and > 500 is great, *** <25% is poor and > 80% is high

The primary parameter derived from molecular docking simulations is the Glide Ligand Efficiency. There are more complementary qualities that are comparable to these. This numerical illustration shows how well the ligand works against certain bacterial proteins. The number of hydrogen bonds formed during interactions between molecules and proteins is estimated by the Glide Hbond measurement [102]. An further metric that clarifies the interactions between chemicals and proteins is the Van der Waals interaction number, or Glide Evdw [101]. Additionally, the Coulomb interactions between proteins and chemicals are quantitatively evaluated via a measure called Glide Ecoul [100]. A quantitative measure derived from the synthesis of several components is the Glide Einternal. The final parameter derived from these computations is this one.

The ADME/T research was conducted to theoretically predict the effects and interactions of the most active molecules inside human metabolism after the biological activities of the compounds were assessed against different proteins [103]. As shown in Table 5, a variety of variables were found throughout this theoretical inquiry.

When developing new drugs, ADME/T (Absorption, Distribution, Metabolism, Elimination/Toxicity) simulations using Schrödinger Maestro software are essential for assessing the pharmacokinetic and toxicological characteristics of compounds [104]. The behavior of a possible chemical in biological systems may be predicted with the use of these simulations. The QikProp module of the Schrödinger program offers a thorough examination of ADME/T parameters.

It is common practice to compute the following parameters and describe them. A molecule’s entire atomic mass is indicated by its molecular weight (MW). As per Lipinski’s “Rule of 5,” a drug candidate is considered successful if its molecular weight is less than 500 daltons of weight. A molecule’s octanol/water partition coefficient is measured by LogP (Hydrophobicity or Lipophilicity), which assesses the molecule’s lipophilicity. Both high and low LogP levels might indicate potential toxicity or inadequate bioavailability. Generally speaking, the ideal LogP value is in the range between 0 and 3. The number of functional groups in a molecule that have the ability to form hydrogen bonds is known as the Hydrogen Bond Donors and Acceptors (HBD/HBA). The solubility and permeability of a molecule across biological membranes are influenced by the quantity of hydrogen bond donors (HBD) and hydrogen bond acceptors (HBA). Generally speaking, it is preferable for drug-like molecules to have five or fewer hydrogen bond donors (HBD) and ten or less hydrogen bond acceptors (HBA). The surface area of a molecule occupied by polar functional groups is known as the total polar surface area, or TPSA. The chemical’s solubility and capacity to pass across biological membranes are related to TPSA. A TPSA of less than 140 Å² indicates favorable bioavailability. The solubility of a molecule in water is measured by Aqueous Solubility (LogS). The drug candidate’s bioavailability and the formulation procedures are impacted by the LogS value [105].

Low solubility molecules often have limited bioavailability. The percentage of a material that binds to plasma proteins is known as plasma protein binding, or PPB. Increased binding to plasma proteins may decrease the drug’s effectiveness and reduce its free concentration [106]. The capacity of a chemical to pass across the blood-brain barrier is known as blood-brain barrier permeability. While decreased BBB permeability may lessen toxicity in systemic therapy, increased BBB permeability is advantageous for medications intended to treat issues with the central nervous system. Metabolic Stability: Forecasts how quickly hepatic enzymes will break down a substance. Increased metabolic stability prolongs the drug’s half-life and improves its therapeutic efficacy. Toxicity Predictions (Hepatotoxicity, HERG Inhibition); the potential cardiotoxicity of HERG channel inhibition is assessed. It is possible to assess the substance’s potential for hepatotoxicity. The translocation of a material across the human intestinal epithelium is replicated by the Caco-2 cell monolayer experiment. Improved intestinal absorption is shown by increased Caco-2 permeability. The percentage of a substance that will be absorbed when administered orally is predicted by its absorption rate. One crucial component of a successful pharmacological medication is a fast rate of absorption [107].

A criterion used to assess a compound’s pharmacological characteristics is Lipinski’s “Rule of Five” (RO5) [108, 109]. These rules, which include Molecular Weight (MW), LogP, Hydrogen Bond Donors (HBD), and Hydrogen Bond Acceptors (HBA), were developed to assess the likelihood that a chemical would have favorable oral bioavailability. In the pharmaceutical development process, lead compounds are identified using a criterion known as the “Rule of Three” (RO3) [110]. This regulation emphasizes the detection of chemically simpler, low molecular weight compounds that are subject to stricter restrictions than RO5. A molecule must fulfill certain requirements in order to be categorized as a lead molecule, under RO3. The criteria of Molecular Weight (MW), LogP, Rotatable Bonds, Hydrogen Bond Donors (HBD), and Hydrogen Bond Acceptors (HBA) are all part of the Rule of Five. Since all parameters are anticipated to meet the necessary requirements, these two parameters’ values are predicted to be zero [111].

Through its computations, it serves as a crucial instrument for evaluating toxicological hazards throughout the drug development process. The likelihood that a proposed chemical would have negative impacts on biological systems is predicted by these simulations. It uses Schrödinger, QikProp, and other modules to evaluate a variety of toxicity criteria. *HERG* (Human Ether-à-go-go-Related Gene) channels are potassium ion channels that control the electrical activity of cardiac myocytes. The first parameter is HERG Channel Inhibition (Cardiotoxicity). Blood-Brain Barrier (BBB) Toxicity is another criteria; substances that may cross the BBB may have harmful effects on the central nervous system. The substrate and inhibition of P-glycoprotein (P-gp) is another metric. P-glycoprotein keeps harmful accumulations at bay by removing chemicals from cells [112].

## Discussion

The main mechanism of action of many clinical anti-cancer agents is to eliminate transformed cells by producing intolerable DNA lesions in the genome. However, in the vast majority of cancer cases, transformed cells develop resistant to the treatment by various mechanisms, including the upregulation of DNA repair proteins, for example those involved in HR [113–115]. A large body of evidence has demonstrated that the natural polyphenolic compound resveratrol can inhibit the tumorigenic growth of various human cancer cells, including breast, ovarian, lung and prostate cancers [116, 117]. Due to its exciting therapeutical potential, resveratrol has recently received a lot of research interest in the context of enhancing the clinical outcomes of personalised treatments [4, 118–121]. In this regard, we describe here resveratrol as a potential chemosensitizer of interest that could be exploited in therapies targeting PARP activity.

Personalised cancer therapy strategies aim to eliminate transformed cells while sparing surrounding healthy cells. The genetic and chemical inhibition of PARP activity have been shown to be specifically cytotoxic to breast and ovarian cancer cells with mutated forms of the tumour suppressors *BRCA1* or *BRCA2* [122, 123]. Current research efforts aiming to increase the clinical efficacy of PARP inhibitors has mostly focused on understanding the functions of novel molecular components involved in HR, which potentially may serve as a synthetic lethal partner for PARP inhibition, since the identification of synthetic lethality between PARP- and HR-driven cellular processes [29, 113–115, 124–128]. Although identification of a HR-deficient condition may enhance the clinical usage of PARP inhibitor therapy, we know that not all breast and ovarian tumour cells have the HR deficiency [86]. Conversely, in some cases, upregulated HR functionality may restrict the activity of HR-targeted therapies such as PARP inhibitors, ultimately causing drug resistance. Indeed, despite successful clinical results, patients may develop PARP inhibitor resistance mostly through the restoration of HR activity [39, 128], highlighting the significance of developing indirect inhibition of HR. There are no known direct inhibitors of the proteins that function in HR. However, recent studies have reported that targeting various cellular pathways can impede the HR mechanism [86, 128, 129].

By performing in vitro cell viability assays, we showed that resveratrol could potentiate the cytotoxicity of olaparib in HR-proficient MCF7 breast cancer cells. The dose- and time-dependent viability assays assessed the combinational synergistic effect of multiple resveratrol and olaparib concentrations in MCF7 cells. Although the interaction between resveratrol and olaparib in MCF7 cells was additive after 24 h of treatment, it became synergistic following 48 h, 72 h and 96 h of treatments. The synergistic combinational cytotoxicity of resveratrol and olaparib was confirmed by multiple cellular assays in MCF7 breast cancer cell lines, indicating that resveratrol could sensitised cancer cells towards olaparib. Our cell culture assays evaluating the long-term effects of the combination of resveratrol and olaparib on the clonogenic survival, tumorigenic formation and migration of breast cancer cells further established that resveratrol enhanced the anti-cancer activity of olaparib. One possible mechanism which could explain why resveratrol render cancer cells sensitive to olaparib is the potential inhibitory effect of resveratrol on DNA repair metabolism. To the extent of our knowledge, Gatz et al. first investigated the effect of resveratrol on DNA DSB repair mechanisms in lymphoblastoid cell lines and revealed that resveratrol directly affects DSB repair even at low concentrations (5 and 30 µM), ultimately inducing an ATM/ATR-dependent DNA damage response [130]. Subsequently, Leon-Galicia et al. performed a comprehensive DNA microarray-based genome-wide screening in order to identify and compare expression profiles of genes in response to resveratrol treatment. Interestingly, they found that the expression of genes such as BRCA2 (Breast Cancer Susceptibility Gene 2), *MRE11 (MRE11 Double Strand Break Repair Nuclease)*, and *RAD51* (RAD51 Recombinase) were downregulated upon high concentrations of resveratrol (150 µM and 250 µM) [20]. Given that MRN (MRE11-RAD50-NBS1) is the essential DNA damage sensor complex [131], by conducting qRT-PCR and Western blotting experiments, they further confirmed the reduced mRNA and protein levels of the MRN components in MCF7 cells especially when challenged by a high dose of resveratrol (250 µM) [20]. In line with our results, the dose- and time-dependent cell viability assays conducted by Leon-Galicia et al. showed the anti-proliferative effects of resveratrol in MCF7 cancer cells [20], which could be explained by the DNA damage accumulation due to impaired DSB repair upon resveratrol treatment. Ruíz et al. further demonstrated that high dose of resveratrol treatment (137 µM) renders cancer stem cells sensitive towards a well-known chemotherapeutic, etoposide, through inhibition of RAD51 expression and activation of apoptosis [22]. Significantly, another study conducted by Leon-Galicia in 2018 showed that resveratrol treatment increases the chemosensitivity of parental and cisplatin-resistant MCF7 cancer cells [19]. Since they previously reported that high doses of resveratrol inhibits mRNA and protein levels of various HR regulators [20], they employed 50 µM and 100 µM resveratrol doses in their research and figured out that resveratrol increases the cytotoxic activity of cisplatin in both chemosensitive and chemoresistant MCF7 cancer cells by downregulating RAD51 mRNA and protein levels and, potentially therefore, causing DNA damage accumulation judged an increased by γ-H2AX formation. Overall, the research identified resveratrol as a potential chemosensitizer acting through the downregulation of essential HR proteins [19].

Furthermore, we conducted immunoblotting experiments and employed antibodies which enabled us to detect and evaluate the expression of biomarkers specific to apoptosis, cell cycle and the activation of DNA damage response. We notably revealed that the formation of cPARP fragments through caspase-3 cleavage, used as an apoptosis marker, was increased by nearly 2-fold in resveratrol and olaparib individual treatment groups compared to untreated control group. However, the cPARP cleavage was tremendously augmented in cells exposed to the highest doses of the compounds applied, suggesting that resveratrol potentiates the cytotoxicity of olaparib and attenuates cancer cell survival by inducing apoptotic cell death. The increase in the cPARP formation was accompanied by a decrease in the cellular level of p21^Waf1/Cip1^, enabling us to rule out the possibility of cell cycle arrest upon co-treatment of resveratrol and olaparib. The cyclin-dependent kinase inhibitor p21Waf1/Cip1 is best known for its growth- and apoptosis-inhibitory functions [132]. However, several reports pointed out that caspase-3-mediated inactivation of p21Waf1/Cip1 protein may prevent cancer cells from growth arrest and enable them to undergoing apoptosis, ultimately leading to chemotherapy-stimulated apoptotic cell death [133–136]. Therefore, considering the cPARP and p21 expression profiles in response to the combination of resveratrol and olaparib, our findings suggest that MCF7 cancer cells is more likely undergo apoptosis. We evaluated whether apoptosis activation in MCF7 cancer cells exposed to the co-treatment of resveratrol and olaparib was occurred as a result of DNA damage accumulation. The phosphorylation of the histone, H2AX, at serine 139 (then named as γH2AX) by the DNA damage kinases (e.g. ATM, ATR) is observed in cells challenged with endogenous or exogenous genotoxic factors which forms DNA DSBs. This is a well-defined significant step for recruiting and localising DNA DSB repair proteins towards the vicinity of damaged chromatin [87]. Given that detection of γH2AX occurrence would enable us to investigate the genomic damage induced by the resveratrol and olaparib combination, we examined the expression of γH2AX (Ser139) as a biomarker for the accumulation of DSBs [87]. Notably, γH2AX (Ser139) was amplified in response to 2.5 µM olaparib and 100 µM resveratrol treatments and the increase was more intense when the co-treatment of resveratrol and olaparib was applied. Although the data propose that the co-treatment of resveratrol and olaparib induces accumulation of DSBs, future studies will need to confirm this by detecting nucleofilament foci formations of γH2AX on chromatin. Indeed, DNA fragmentation and AO/EB assays we conducted also confirmed that apoptosis occurs in MCF7 cancer cells upon the combination of resveratrol (100 µM) and olaparib (2.5 µM) treatments. Consequently, olaparib disables the single-strand DNA repair mechanism and causes the accumulation DNA single-strand breaks, which are then converted to double-strand DNA breaks mostly during replication. On one hand, cancer cells with fully functional HR activity (or overactivated HR activity) can effectively repair the lethal DSBs and obtain damage-repair resistance granting a long-term cancer cell survival. On the other hand, resveratrol could inhibit HR functionality, and therefore, increases the cytotoxic capacity of olaparib.

Consistent with our conclusion, a very recent report published by Bellare and Patro in 2022 showed that resveratrol effectively augmented anti-neoplastic effects of talazoparib, another clinically approved PARP inhibitor, by inducing p38-MAPK-mediated apoptotic cell death in MCF7 and MDA-MB-231 breast cancer cells [137]. They performed immunofluorescence analysis by co-labelling 53BP1 and γH2AX in order to directly assess DSB foci formations, revealing that cells treated with both resveratrol and talazoparib accumulated DSB foci formations. Furthermore, they employed GFP-reporter assays assessing the efficacy of the HR activity in MCF7 cells and figured out that the combination of resveratrol and olaparib effectively diminished the HR activity, overall supporting our hypothesis that resveratrol increases the olaparib-sensitivity of breast cancer cells due to impaired HR activity. Our experiments align with a recent report by Sinha et al. (2022) demonstrating that olaparib enhances resveratrol-mediated apoptosis in breast cancer cells [138]. While our findings and those of others [22, 139, 140] indicated that at least 100 µM resveratrol was required to eliminate half of the cells after 24 h of treatment, Sinha et al. reported an IC50 of 25 µM for resveratrol in MCF7 cells [138]. Additionally, the IC50 value of olaparib in MCF7 cells was relatively lower compared to our findings and other studies [141–143]. Despite differences in applied concentrations, our results further support the idea that resveratrol treatment enhances the cytotoxicity of olaparib by inducing DNA damage and activating apoptosis in MCF7 breast cancer cells. Building on recent reports [19, 20, 22], we hypothesised that resveratrol treatment restricts the HR DNA double-strand break repair capacity, thereby sensitising cancer cells to PARP inhibition. Indeed, a very recent report revealed that the combination of resveratrol and olaparib efficiently disrupts the HR repair pathway in breast cancer cells by inhibiting chromatin relaxation [144]. Subsequent investigations will contribute to elucidating the precise mechanisms through which resveratrol enhances the cytotoxic effects of olaparib in *BRCA1/2*-effective breast cancer cells.

As a result of theoretical calculations, the quantum chemical parameters of the molecules Resveratrol, olaparib, and resveratrol and olaparib combinations were first calculated with the Gaussian software program. The activities of the molecules in a pure and isolated environment were compared with these quantum chemical parameters. In general, it was seen that the molecule with resveratrol and olaparib combinations had higher activity.

Then, the formation of resveratrol and olaparib combinations was theoretically examined. When the numerical values of the calculated ∆G, ∆H and ∆E parameters were examined, it was seen that the ∆G values were negative at the M062X level and positive at the B3LYP and HF levels. In this case, it shows that the formation of resveratrol and olaparib combinations occurred spontaneously.

On the other hand, the activities of the molecules against breast cancer proteins were compared. Here, the activities of the molecules as a result of their interactions with proteins were compared. In the comparisons made, it was seen that the resveratrol and olaparib combinations molecule had a docking score value of −4.22 against the 1JNX protein. On the other hand, the highest activity against the 1A52 protein was the resveratrol molecule with a docking score value of −8.10. The fact that the molecules have high activity does not mean that they will be a good drug. Therefore, according to the ADME/T analysis results, although the resveratrol and olaparib combinations molecule seems to be a suitable drug according to the key-lock model, it is seen that it has quite high values in terms of both molar mass and volume. When we look at the resveratrol and olaparib molecules, it is seen that they provide all the ADME/T parameters examined in general. Since the Rule of Five and Rule of Three parameters are also zero, it is thought to be an ideal drug molecule.

In summary, the preclinical evidence presented in our study reinforces the notion that resveratrol holds promise as a therapeutic agent to augment the anti-cancer effects of the PARP inhibitor olaparib in HR-proficient breast cancer cells. Thus, our findings contribute valuable insights toward the development of novel therapeutic strategies aimed at targeting PARP activity, with the overarching goal of enhancing the efficacy of PARP inhibitor therapies.

## Conclusion

The current study underscores the therapeutic promise of combining resveratrol with olaparib in MCF7 breast cancer cells. Targeting PARP activity specifically induces cytotoxicity in cancer cells with HR deficiency. While several PARP inhibitors have been developed and effectively utilized in treating cancer patients with HR-deficient profiles in clinical settings, some patients may develop drug resistance over time, either de novo or acquired, by restoring HR activity. This resistance limits the clinical utility of PARP inhibitor therapy. Therefore, there is a clinical need to indirectly inhibit HR to enhance the efficacy of PARP inhibitor therapies. Previous studies have demonstrated that resveratrol treatment disrupts HR functionality in breast cancer cells. Building on this observation, our study provides further evidence that resveratrol enhances the anti-cancer potential of olaparib in breast cancer cells. As a result of theoretical calculations, the activities of the molecules Resveratrol, olaparib, and resveratrol and olaparib combinations were compared with both quantum chemical calculations and molecular docking calculations. Finally, the drug properties of the molecules were examined by ADME/T calculations. Consequently, our findings suggest that resveratrol-mediated HR inhibition could be a novel therapeutic strategy to consider for personalized cancer therapies involving PARP inhibitors in HR-proficient cancer cells. Future studies should investigate the molecular mechanisms underpinning the resveratrol–olaparib synergy, particularly focusing on DNA repair pathway modulation, PARP–protein interactome alterations, and potential effects on HR- or *BRCA*-deficient cellular models. In vivo validation using xenograft or patient-derived tumor models, alongside comprehensive pharmacokinetic, ADME/T, and dose-optimization analyses, will be essential to determine the translational potential of this combination for breast cancer therapy.

## References

[1] Hashem S, Ali TA, Akhtar S, Nisar S, Sageena G, Ali S et al (2022) Targeting cancer signaling pathways by natural products: exploring promising anti-cancer agents. Biomed Pharmacother 150:113054. 10.1016/j.biopha.2022.113054 35658225 10.1016/j.biopha.2022.113054

[2] Naeem A, Hu P, Yang M, Zhang J, Liu Y, Zhu W et al (2022) Natural products as anticancer agents: current status and future perspectives. Molecules 27:1–64 10.3390/molecules27238367 PMC9737905 36500466

[3] Kansu G, Ozturk N, Karagac MS, Yesilkent EN, Ceylan H (2025) The interplay between doxorubicin chemotherapy, antioxidant system, and cardiotoxicity: unrevealing of the protective potential of tannic acid. Biotechnol Appl Biochem 72(1):75–85. 10.1002/bab.2648 39099314 10.1002/bab.2648 PMC11798539

[4] Singh AP, Singh R, Verma SS, Rai V, Kaschula CH, Maiti P et al (2019) Health benefits of resveratrol: evidence from clinical studies. Med Res Rev 39(5):1851–91 30741437 10.1002/med.21565

[5] Galiniak S, Aebisher D, Bartusik-Aebisher D (2019) Health benefits of resveratrol administration. Acta Biochim Pol 66(1):13–21 30816367 10.18388/abp.2018_2749

[6] Pervaiz S Chemotherapeutic potential of the chemopreventive phytoalexin resveratrol. Drug Resist Updat [Internet]. 2004 Dec [cited 2019 Jul 25];7(6):333–44. Available from: http://www.ncbi.nlm.nih.gov/pubmed/15790544 10.1016/j.drup.2004.11.001 15790544

[7] Ko JH, Sethi G, Um JY, Shanmugam MK, Arfuso F, Kumar AP et al (2017) The role of Resveratrol in cancer therapy. Int J Mol Sci 18(12):1–36 10.3390/ijms18122589 PMC5751192 29194365

[8] Carter LG, D’Orazio JA, Pearson KJ (2014) Resveratrol and cancer: focus on in vivo evidence. Endocr Relat Cancer 21(3):R209 24500760 10.1530/ERC-13-0171 PMC4013237

[9] Rauf A, Imran M, Butt MS, Nadeem M, Peters DG, Mubarak MS (2018) Resveratrol as an anti-cancer agent: a review. Crit Rev Food Sci Nutr 58(9):1428–1447 28001084 10.1080/10408398.2016.1263597

[10] Jang M, Cai L, Udeani GO, Slowing KV, Thomas CF, Beecher CWW et al (1997) Cancer chemopreventive activity of resveratrol, a natural product derived from grapes. Science 275(5297):218–20 8985016 10.1126/science.275.5297.218

[11] Bhat KPL, Lantvit D, Christov K, Mehta RG, Moon RC, Pezzuto JM (2001) Estrogenic and antiestrogenic properties of resveratrol in mammary tumor models. Cancer Res 61(20):7456–7463 11606380

[12] Garvin S, Öllinger K, Dabrosin C (2006) Resveratrol induces apoptosis and inhibits angiogenesis in human breast cancer xenografts in vivo. Cancer Lett 231(1):113–122 16356836 10.1016/j.canlet.2005.01.031

[13] Li Y, Liu J, Liu X, Xing K, Wang Y, Li F et al (2006) Resveratrol-induced cell Inhibition of growth and apoptosis in MCF7 human breast cancer cells are associated with modulation of phosphorylated Akt and caspase-9. Appl Biochem Biotechnol 135(3):181–192 17299206 10.1385/abab:135:3:181

[14] Casanova F, Quarti J, Ferraz Da Costa DC, Ramos CA, Da Silva JL, Fialho E (2012) Resveratrol chemosensitizes breast cancer cells to melphalan by cell cycle arrest. J Cell Biochem 113(8):2586–96 22415970 10.1002/jcb.24134

[15] Hu S, Li X, Xu R, Ye L, Kong H, Zeng X et al (2016) The synergistic effect of resveratrol in combination with cisplatin on apoptosis via modulating autophagy in A549 cells. Acta Biochim Biophys Sin 48(6):528–35 27084520 10.1093/abbs/gmw026 PMC4913517

[16] Mirzapur P, Khazaei MR, Moradi MT, Khazaei M (2018) Apoptosis induction in human breast cancer cell lines by synergic effect of raloxifene and resveratrol through increasing proapoptotic genes. Life Sci 205:45–53. 10.1016/j.lfs.2018.04.035 29705353 10.1016/j.lfs.2018.04.035

[17] Muhanmode Y, Wen MK, Maitinuri A, Shen G (2022) Curcumin and resveratrol inhibit chemoresistance in cisplatin-resistant epithelial ovarian cancer cells via targeting P13K pathway. Hum Exp Toxicol 41:096032712210959 10.1177/09603271221095929 35722665

[18] Zhang W, Jiang H, Chen Y, Ren F (2019) Resveratrol chemosensitizes adriamycin-resistant breast cancer cells by modulating miR-122-5p. J Cell Biochem 120(9):16283–16292 31155753 10.1002/jcb.28910

[19] Leon–Galicia I, Diaz–Chavez J, Albino–Sanchez ME, Garcia–Villa E, Bermudez–Cruz R, Garcia–Mena J et al Resveratrol decreases Rad51 expression and sensitizes cisplatin–resistant MCF–7 breast cancer cells. Oncol Rep [Internet]. 2018 Mar 27 [cited 2019 Jul 25];39(6):3025–33. Available from: http://www.ncbi.nlm.nih.gov/pubmed/29620223 10.3892/or.2018.6336 29620223

[20] Leon-Galicia I, Diaz-Chavez J, Garcia-Villa E, Uribe-Figueroa L, Hidalgo-Miranda A, Herrera LA et al Resveratrol induces downregulation of DNA repair genes in MCF-7 human breast cancer cells. Eur J Cancer Prev [Internet]. 2013 Jan 1 [cited 2019 Jul 25];22(1):11–20. Available from: http://www.ncbi.nlm.nih.gov/pubmed/22644231 10.1097/CEJ.0b013e328353edcb 22644231

[21] Syed A, Tainer JA (2018) The MRE11-RAD50-NBS1 complex conducts the orchestration of damage signaling and outcomes to stress in DNA replication and repair. Annu Rev Biochem 87:263–294 29709199 10.1146/annurev-biochem-062917-012415 PMC6076887

[22] Ruíz G, Valencia-González HA, León-Galicia I, García-Villa E, García-Carrancá A, Gariglio P (2018) Inhibition of RAD51 by siRNA and resveratrol sensitizes cancer stem cells derived from HeLa cell cultures to apoptosis. Stem Cells Int. 10.1155/2018/2493869 29681946 10.1155/2018/2493869 PMC5846439

[23] Krishnakumar R, Kraus WL (2010) The PARP side of the nucleus: molecular actions, physiological outcomes, and clinical targets. Mol Cell 39(1):8–24. 10.1016/j.molcel.2010.06.017 20603072 10.1016/j.molcel.2010.06.017 PMC2923840

[24] Pines A, Vrouwe MG, Marteijn JA, Typas D, Luijsterburg MS, Cansoy M et al (2012) PARP1 promotes nucleotide excision repair through DDB2 stabilization and recruitment of ALC1. J Cell Biol 199(2):235–49 23045548 10.1083/jcb.201112132 PMC3471223

[25] Zhao Q, Lan T, Su S, Rao Y (2019) Induction of apoptosis in MDA-MB-231 breast cancer cells by a PARP1-targeting PROTAC small molecule. Chem Commun 55(3):369–372 10.1039/c8cc07813k 30540295

[26] Rose M, Burgess JT, O’Byrne K, Richard DJ, Bolderson E (2020) PARP inhibitors: clinical relevance, mechanisms of action and tumor resistance. Front Cell Dev Biol 8(564601):1–22 33015058 10.3389/fcell.2020.564601 PMC7509090

[27] Morales JC, Li L, Fattah FJ, Dong Y, Bey EA, Patel M et al (2014) Review of poly (ADP-ribose) polymerase (PARP) mechanisms of action and rationale for targeting in cancer and other diseases. Crit Rev Eukaryot Gene Expr 24(1):15–28 24579667 10.1615/critreveukaryotgeneexpr.2013006875 PMC4806654

[28] Lord CJ, Ashworth A (2017) PARP inhibitors: synthetic lethality in the clinic. Sci (80-) 355(6330):1152–1158 10.1126/science.aam7344 PMC6175050 28302823

[29] Topatana W, Juengpanich S, Li S, Cao J, Hu J, Lee J et al (2020) Advances in synthetic lethality for cancer therapy: cellular mechanism and clinical translation. J Hematol Oncol 13(1):1–22 32883316 10.1186/s13045-020-00956-5 PMC7470446

[30] Erdogan MK, Usca AB (2025) Gallic acid enhances Olaparib-induced cell death and attenuates Olaparib resistance in human osteosarcoma U2OS cell line. Curr Issues Mol Biol 47(2):104. 10.3390/cimb47020104 39996825 10.3390/cimb47020104 PMC11854715

[31] Li S, Topatana W, Juengpanich S, Cao J, Hu J, Zhang B et al (2020) Development of synthetic lethality in cancer: molecular and cellular classification. Signal Transduct Target Ther. 10.1038/s41392-020-00358-6 33077733 10.1038/s41392-020-00358-6 PMC7573576

[32] Lord CJ, Tutt ANJ, Ashworth A (2015) Synthetic Lethality and Cancer Therapy: Lessons Learned from the Development of PARP Inhibitors. Annu Rev Med [Internet]. Jan 14 [cited 2018 Dec 13];66(1):455–70. Available from: http://www.ncbi.nlm.nih.gov/pubmed/25341009 10.1146/annurev-med-050913-022545 25341009

[33] Sachdev E, Tabatabai R, Roy V, Rimel BJ, Mita MM (2019) PARP inhibition in cancer: an update on clinical development. Target Oncol 14(6):657–679 31625002 10.1007/s11523-019-00680-2

[34] Walsh C (2018) Targeted therapy for ovarian cancer: the rapidly evolving landscape of PARP inhibitor use. Minerva Ginecol 70(2):150–170 28994564 10.23736/S0026-4784.17.04152-1

[35] The Food and Drug Administration (FDA) FDA approves olaparib for HRR gene-mutated metastatic castration-resistant prostate cancer [Internet]. 2020 [cited 2022 Sep 1]. Available from: https://www.fda.gov/drugs/resources-information-approved-drugs/fda-approves-olaparib-hrr-gene-mutated-metastatic-castration-resistant-prostate-cancer

[36] The Food and Drug Administration (FDA) FDA approves olaparib for gBRCAm metastatic pancreatic adenocarcinoma [Internet]. 2019 [cited 2022 Sep 1]. Available from: https://www.fda.gov/drugs/resources-information-approved-drugs/fda-approves-olaparib-gbrcam-metastatic-pancreatic-adenocarcinoma

[37] The Food and Drug Administration (FDA) FDA approves olaparib for germline BRCA-mutated metastatic breast cancer [Internet]. 2018 [cited 2020 May 1]. Available from: https://www.fda.gov/drugs/resources-information-approved-drugs/fda-approves-olaparib-germline-brca-mutated-metastatic-breast-cancer

[38] The Food and Drug Administration (FDA) (2022) FDA approves olaparib for adjuvant treatment of high-risk early breast cancer

[39] D’Andrea AD (2018) Mechanisms of PARP inhibitor sensitivity and resistance. DNA Repair (Amst) 71:172–176 30177437 10.1016/j.dnarep.2018.08.021

[40] Noordermeer SM, van Attikum H (2019) PARP inhibitor resistance: a tug-of-war in BRCA-Mutated cells. Trends Cell Biol 29(10):820–34 31421928 10.1016/j.tcb.2019.07.008

[41] Dréan A, Lord CJ, Ashworth A (2016) PARP inhibitor combination therapy. Crit Rev Oncol Hematol 108:73–85. 10.1016/j.critrevonc.2016.10.010 27931843 10.1016/j.critrevonc.2016.10.010

[42] Allah Abderrazzak El Moutaouakil Ala et al (2025) Novel thiohydantoin derivatives: design, synthesis, spectroscopic characterization, crystal structure, SAR, DFT, molecular docking, pharmacological and toxicological activities. J Mol Struct 1335:141995

[43] Karatas Halis et al (2025) Alzheimer’s disease drug design by synthesis, characterization, enzyme inhibition, in silico, SAR analysis and MM-GBSA analysis of Schiff bases derivatives. Korean J Chem Eng. 10.1007/s11814-025-00433-0

[44] Eddhimi Ayoub et al (2025) Growth, molecular docking, Hirshfeld surface analysis and first-principles investigation on the structural, morphological and mechanical properties of the OIH hybrid: C2H8N4S22+· 2HSO4 − under pressure. J Mol Struct 1324:140809

[45] Tüzün Burak (2025) Evaluation of cytotoxicity, chemical composition, antioxidant potential, apoptosis relationship, molecular docking, and MM-GBSA analysis of *Rumex crispus* leaf extracts. J Mol Struct 1323:140791

[46] Poustforoosh Alireza et al (2024) Tracing the pathways and mechanisms involved in the anti-breast cancer activity of glycyrrhizin using bioinformatics tools and computational methods. J Biomol Struct Dyn 42(2):819–833 37042955 10.1080/07391102.2023.2196347

[47] Becke Axel D (1992) Density-functional thermochemistry. I. The effect of the exchange‐only gradient correction. J Chem Phys 96(3):2155–2160

[48] Vautherin D, Brink DM (1972) T. hartree-Fock calculations with skyrme’s interaction. I. Spherical nuclei. Phys Rev C 5(3):626

[49] Hohenstein EG, Chill ST, Sherrill C, David (2008) Assessment of the performance of the M05 – 2X and M06 – 2X exchange-correlation functionals for noncovalent interactions in biomolecules. J Chem Theory Comput 4(12):1996–2000 26620472 10.1021/ct800308k

[50] Tanenbaum David M. et al (1998) Crystallographic comparison of the estrogen and progesterone receptor’s ligand binding domains. Proc Natl Acad Sci U S A 95(11):5998–6003 9600906 10.1073/pnas.95.11.5998 PMC27574

[51] Williams R. Scott, Green Ruth, Glover JN Mark (2001) Crystal structure of the BRCT repeat region from the breast cancer-associated protein BRCA1. Nat Struct Biol 8(10):838–842 11573086 10.1038/nsb1001-838

[52] Erdogan MK, Gundogdu R et al (2025) Deciphering the anticancer, antioxidant, enzyme inhibition potentials, and phytochemical compositions of some endemic *Centaurea* species. Chem Biodivers 22(8):e202403185. 10.1002/cbdv.202403185 40178386 10.1002/cbdv.202403185

[53] Chou T-C, Talalay P Quantitative analysis of dose-effect relationships: the combined effects of multiple drugs or enzyme inhibitors. Adv Enzyme Regul [Internet]. 1984 Jan 1 [cited 2019 Sep 20];22:27–55. Available from: https://www.sciencedirect.com/science/article/abs/pii/0065257184900074 10.1016/0065-2571(84)90007-4 6382953

[54] Erdogan MK, Ozer G (2025) Synergistic anticancer effects of bleomycin and hesperidin combination on A549 non-small cell lung cancer cells: Antiproliferative, Apoptotic, Anti-Angiogenic, and autophagic insights. Pharmacol 18(2):254 10.3390/ph18020254 PMC11859711 40006067

[55] Parker C, Chambers AC, Flanagan DJ, Ho JWY, Collard TJ, Ngo G et al (2022) BCL-3 loss sensitises colorectal cancer cells to DNA damage by targeting homologous recombination. DNA Repair 115:103331. 10.1016/j.dnarep.2022.103331 35468497 10.1016/j.dnarep.2022.103331 PMC10618080

[56] Gök Ö, Aslan A, Erdoğan MK, Uslu H, Toy Y, … Gundogdu R. The effect of PARP and PLK1 dual inhibition on the expression of important protein signaling pathways, DNA damage,and molecular docking scores against MCF-7 and MDA-MB-231 breast cancer cell lines.Irish J. of Med. Sci (1971-). 2025: 1–22. 10.1007/s11845-025-04127-8 41233610

[57] Repetto G, del Peso A, Zurita JL (2008) Neutral red uptake assay for the estimation of cell viability/ cytotoxicity. Nat Protoc 3(7):1125–1131 18600217 10.1038/nprot.2008.75

[58] Erdogan MK, Toy Y, Gundogdu R, Gecibesler IH, Sever A, Yapar Y, Behcet L, Zengin G (2025) Assessment of cytotoxic, apoptotic, enzyme inhibitory, and antioxidant properties, and phytochemical characterization of ethanolic extract from Cionura erecta. Food Biosci 65:106082

[59] Gundogdu R, Erdogan MK, Ditsiou A, Spanswick V, Garcia-Gomez JJ, Hartley JA et al (2021) hMOB2 deficiency impairs homologous recombination-mediated DNA repair and sensitises cancer cells to PARP inhibitors. Cell Signal 87:110106. 10.1016/j.cellsig.2021.110106 34363951 10.1016/j.cellsig.2021.110106 PMC8514680

[60] Erdogan MK, Sever A, Gundogdu R et al (2025) Verbascum gimgimense an Endemic Turkish Plant: Evaluation of In Vitro Anticancer, Antioxidant, Enzyme Inhibitory Activities, and Phytochemical Profile. Cell Biochem. and Funct. ; 42(8): e70023. Available from: 10.1002/cbf.70023 10.1002/cbf.70023 39632482

[61] Bettoun A, Joffre C, Zago G, Surdez D, Vallerand D, Gundogdu R et al (2016) Mitochondrial clearance by the STK38 kinase supports oncogenic Ras-induced cell transformation. Oncotarget 7(28):44142–44160 27283898 10.18632/oncotarget.9875 PMC5190085

[62] Kasibhatla S, Amarante-Mendes GP, Finucane D, Brunner T, Bossy-Wetzel E, Green DR (2006) Analysis of DNA fragmentation using agarose gel electrophoresis. Cold Spring Harb Protoc 2006(1):pdb.prot4429 10.1101/pdb.prot4429 22485764

[63] Erdogan MK, Gundogdu R, Toy Y, Gecibesler IH, Yapar Y, Behcet L, Zengin G (2024) Comparison of anticancer, antioxidant, enzyme inhibitory effects and phytochemical contents between edible lettuce (*Lactuca sativa*) and a new wild species (*Lactuca anatolica*). Chem Biodivers 21(9):e202400552 38958194 10.1002/cbdv.202400552

[64] Dennington R, Keith TA, Millam JM (2016) GaussView 6.0. 16, semichem inc. Shawnee Mission KS USA, 143–150

[65] Frisch MJ et al (2009) Uranyl extraction by N, N-dialkylamide ligands studied by static and dynamic DFT simulations. Gaussian 9(9):227 10.1039/c4dt02443e 25412447

[66] Medetalibeyoğlu Hilal et al (2025) Synthesis, design, and cholinesterase inhibitory activity of novel 1, 2, 4-triazole Schiff bases: a combined experimental and computational approach. Int J Biol Macromol 306:141350 39986523 10.1016/j.ijbiomac.2025.141350

[67] Schrödinger Release 2022-4 (2022) Maestro, Schrödinger, LLC, New York, NY

[68] Schrödinger Release 2022-4 (2022) Protein Preparation Wizard; Epik, Schrödinger. LLC, New York, NY

[69] Impact Schrödinger, New York LLC, Prime NY (2022) Schrödinger, LLC, New York, NY

[70] Schrödinger (2022) Release 2022-4: LigPrep, Schrödinger. LLC, New York, NY

[71] Shahzadi I et al (2022) Repositioning of Acefylline as anti-cancer drug: synthesis, anticancer and computational studies of azomethines derived from Acefylline tethered 4-amino-3-mercapto-1, 2, 4-triazole. PLoS One 17(12):e0278027 36520942 10.1371/journal.pone.0278027 PMC9754256

[72] El Faydy M et al (2024) Synthesis, biological properties, and molecular docking study of novel 1, 2, 3-triazole-8-quinolinol hybrids. ACS Omega 9(23):25395–25409 38882066 10.1021/acsomega.4c03906 PMC11170742

[73] Schrödinger (2022) Release 2022-4: QikProp, Schrödinger, LLC, New York, NY

[74] Scarcello E, Lambremont A, Vanbever R, Jacques PJ, Lison D (2020) Mind your assays: misleading cytotoxicity with the WST-1 assay in the presence of manganese. PLoS One 15(4):1–14. 10.1371/journal.pone.0231634 10.1371/journal.pone.0231634 PMC7161962 32298350

[75] Elstrodt F, Hollestelle A, Nagel JHA, Gorin M, Wasielewski M, Van Den Ouweland A et al (2006) BRCA1 mutation analysis of 41 human breast cancer cell lines reveals three new deleterious mutants. Cancer Res 66(1):41–5 16397213 10.1158/0008-5472.CAN-05-2853

[76] Kao J, Salari K, Bocanegra M, Choi Y La, Girard L, Gandhi J et al (2009) Molecular profiling of breast cancer cell lines defines relevant tumor models and provides a resource for cancer gene discovery. PLoS One. 10.1371/journal.pone.0006146 19582160 10.1371/journal.pone.0006146 PMC2702084

[77] Cifone MA, Fidler IJ (1980) Correlation of patterns of anchorage-independent growth with in vivo behavior of cells from a murine fibrosarcoma. Proc Natl Acad Sci U S A 77(2 II):1039–43 6928659 10.1073/pnas.77.2.1039 PMC348419

[78] Shin SI, Freedman VH, Risser R, Pollack R (1975) Tumorigenicity of virus transformed cells in nude mice is correlated specifically with anchorage independent growth in vitro. Proc Natl Acad Sci U S A 72(11):4435–4439 172908 10.1073/pnas.72.11.4435 PMC388736

[79] Wang X, Decker CC, Zechner L, Krstin S, Wink M (2019) In vitro wound healing of tumor cells: inhibition of cell migration by selected cytotoxic alkaloids. BMC Pharmacol Toxicol 20(1):1–12 30626448 10.1186/s40360-018-0284-4 PMC6327619

[80] Kauanova S, Urazbayev A, Vorobjev I (2021) The frequent sampling of wound scratch assay reveals the opportunity window for quantitative evaluation of cell motility-impeding drugs. Front Cell Dev Biol 9(March):1–14 10.3389/fcell.2021.640972 PMC7991799 33777948

[81] Mashimo M, Onishi M, Uno A, Tanimichi A, Nobeyama A, Mori M et al (2021) The 89-kDa PARP1 cleavage fragment serves as a cytoplasmic PAR carrier to induce AIF-mediated apoptosis. J Biol Chem 296:100046. 10.1074/jbc.RA120.014479 33168626 10.1074/jbc.RA120.014479 PMC7948984

[82] Duriez PJ, Shah GM (1997) Cleavage of poly(ADP-ribose) polymerase: a sensitive parameter to study cell death. Biochem Cell Biol 75(4):337–349 9493956

[83] Kulaberoglu Y, Gundogdu R, Hergovich A (2016) The Role of p53/p21/p16 in DNA-Damage Signaling and DNA Repair. Genome Stability. pp 243–53

[84] Goulooze SC, Cohen AF, Rissmann R, Olaparib Br J Clin Pharmacol [Internet]. 2016 Jan [cited 2018 Dec 13];81(1):171–3. Available from: http://www.ncbi.nlm.nih.gov/pubmed/26344419 10.1111/bcp.12761 PMC4693566 26344419

[85] Giovannini S, Weller MC, Repmann S, Moch H, Jiricny J (2019) Synthetic lethality between BRCA1 deficiency and poly(ADP-ribose) polymerase inhibition is modulated by processing of endogenous oxidative DNA damage. Nucleic Acids Res 47(17):9132–9143 31329989 10.1093/nar/gkz624 PMC6753488

[86] Pilié PG, Gay CM, Byers LA, O’Connor MJ, Yap TA (2019) PARP inhibitors: extending benefit beyond BRCA-mutant cancers. Clin Cancer Res 25(13):3759–3771 30760478 10.1158/1078-0432.CCR-18-0968

[87] Kuo LJ, Yang LX (2008) γ-H2AX- A novel biomaker for DNA double-strand breaks. Vivo (Brooklyn) 22(3):305–309 18610740

[88] Akkus Musa et al (2025) Phenolic compounds: investigating their anti-carbonic anhydrase, anti-cholinesterase, anticancer, anticholinergic, and antiepileptic properties through molecular docking, MM-GBSA, and dynamics analyses. Korean J Chem Eng 42(5):1149–1168

[89] Mbomyufanyi Divine et al (2024) A cadmium-thiocyanate coordination polymer with bridging 2-aminopyridine: synthesis, characterization DFT studies, Hirshfeld surface analysis and docking studies. J Mol Struct 1318:139292

[90] Manap Sevda et al (2024) Synthesis, molecular modeling investigation, molecular dynamic and ADME prediction of some novel Mannich bases derived from 1, 2, 4-triazole, and assessment of their anticancer activity. J Biomol Struct Dyn 42(21):11916–11930 37840297 10.1080/07391102.2023.2265501

[91] Kaya Savaş et al (2016) Determination of corrosion inhibition effects of amino acids: quantum chemical and molecular dynamic simulation study. J Taiwan Inst Chem Eng 58:528–535

[92] Myroslava Ohloblina et al (2024) Molecular descriptors and in silico studies of 4-((5-(decylthio)-4-methyl-4n-1, 2, 4-triazol-3-yl) methyl) morpholine as a potential drug for the treatment of fungal pathologies. Comput Biol Chem 113:108206 39265461 10.1016/j.compbiolchem.2024.108206

[93] Schrader Tim, Khanifaev Jamoliddin, Perlt Eva (2023) Koopmans’ theorem for acidic protons. Chem Commun 59(93):13839–13842 10.1039/d3cc04304e 37921279

[94] Pearson RG (1963) Hard and soft acids and bases. J Am Chem Soc 85(22):3533–3539

[95] Parr RG, Chattaraj PK (1991) Principle of maximum hardness. J Am Chem Soc 113(5):1854–1855

[96] Aksu Aysun et al (2023) Immobilization of pectinase on chitosan-alginate-clay composite beads: experimental, DFT and molecular docking studies. J Mol Liq 390:122947

[97] Eyupoglu Volkan et al (2024) Biosorption of dye crystal violet on *Tragopogon* sp. leaf powder: equilibrium, kinetics, thermodynamics, and DFT calculations. J Mol Liq 398:124226

[98] Jiménez JS, Benítez MaríaJ (2024) Gibbs free energy and enthalpy–entropy compensation in protein–ligand interactions. Biophysica 4(2):298–309

[99] Çi̇çek Semra et al (2024) A study on insecticidal activity of the fennel (*Foeniculum vulgare*) essential oil and its nanoemulsion against stored product pests and molecular docking evaluation. Ind Crops Prod 222:119859

[100] Kapancik S et al (2024) Chemical composition, cytotoxicity, and molecular Docking analyses of Thuja orientalis extracts. J Mol Struct 1318:139279

[101] Barghady Najoua et al (2024) Design, synthesis, characterization, and theoretical calculations, along with in silico and in vitro antimicrobial proprieties of new isoxazole-amide conjugates. Open Chem 22(1):20240109

[102] Goswami Ashis Kumar et al (2024) Integrative in silico evaluation of the antiviral potential of terpenoids and its metal complexes derived from *Homalomena aromatica* based on main protease of SARS-CoV-2. Open Chem 22(1):20240085

[103] Medetalibeyoğlu Hilal et al (2025) Novel Schiff bases: synthesis, characterization, bioactivity, cytotoxicity, and computational evaluations. Polycycl Aromat Compd 45(4):541–559

[104] Tapera Michael et al (2024) Novel 1, 2, 4-triazole-maleamic acid derivatives: synthesis and evaluation as anticancer agents with carbonic anhydrase inhibitory activity. J Mol Struct 1313:138680

[105] Khalilov Ali N. et al (2024) Synthesis, crystal structure, Hirshfeld surface analyses, and DFT studies of (S)-2-(3, 5-di-tert-butyl-4-hydroxyphenyl)-3, 3-diethoxy-1-phenylpropan-1-one. J Mol Struct 1313:138652

[106] Alishba Exploring Benzo [b][1, 4] Thiazine Derivatives: Multitarget Inhibition, Structure–Activity Relationship, Molecular Docking, and, Analysis ADMET et al (2024) *ChemistrySelect*, 9.38: e202404087

[107] Çelik M, Safa et al (2024) Removal of Safranin O from wastewater using streptomyces Griseobrunneus dead biomass and in Silico calculations. Biomass Convers Biorefinery 1420:25873–25884

[108] Lipinski CA (2004) Lead-and drug-like compounds: the rule-of-five revolution. Drug Discovery Today: Technol 1(4):337–341 10.1016/j.ddtec.2004.11.007 24981612

[109] Lipinski Christopher A. et al (1997) Experimental and computational approaches to estimate solubility and permeability in drug discovery and development settings. Adv Drug Deliv Rev 23(1–3):3–25 10.1016/s0169-409x(00)00129-0 11259830

[110] Jorgensen WL, Duffy EM (2002) Prediction of drug solubility from structure. Adv Drug Deliv Rev 543:355–366 10.1016/s0169-409x(02)00008-x 11922952

[111] Güleç Özcan et al (2024) Peripheral (E)-2‐[(4‐hydroxybenzylidene)‐3, 4‐dihydronaphthalen‐1 (2H)‐one)]‐coordinated phthalocyanines with improved enzyme inhibition properties and photophysicochemical behaviors. Arch Pharm (Weinheim) 357(9):2400209 10.1002/ardp.202400209 38838335

[112] Zahirović Adnan et al (2024) Hydrazone-flavonol based oxidovanadium (V) complexes: synthesis, characterization and antihyperglycemic activity of chloro derivative in vivo. J Inorg Biochem 258:112637 38876026 10.1016/j.jinorgbio.2024.112637

[113] Huang A, Garraway LA, Ashworth A, Weber B (2020) Synthetic lethality as an engine for cancer drug target discovery. Nat Rev Drug Discov 19(1):23–38. 10.1038/s41573-019-0046-z 31712683 10.1038/s41573-019-0046-z

[114] Jackson SP, Helleday T, Drugging DNA repair. Science (80-) [Internet]. 2016 Jun 3 [cited 2018 Sep 27];352(6290):1178–9. Available from: http://www.ncbi.nlm.nih.gov/pubmed/27257245 10.1126/science.aab0958 27257245

[115] Nickoloff JA, Jones D, Lee SH, Williamson EA, Hromas R (2017) Drugging the cancers addicted to DNA repair. J Natl Cancer Inst 109(11):1–11 10.1093/jnci/djx059 PMC5436301 28521333

[116] Gupta SC, Kannappan R, Reuter S, Kim JH, Aggarwal BB (2011) Chemosensitization of tumors by resveratrol. Ann N Y Acad Sci 1215(1):150–160 21261654 10.1111/j.1749-6632.2010.05852.x PMC3060406

[117] Berman AY, Motechin RA, Wiesenfeld MY, Holz MK (2017) The therapeutic potential of resveratrol: a review of clinical trials. NPJ Precis Oncol. 10.1038/s41698-017-0038-6 28989978 10.1038/s41698-017-0038-6 PMC5630227

[118] Atanasov AG, Waltenberger B, Pferschy-Wenzig EM, Linder T, Wawrosch C, Uhrin P et al (2015) Discovery and resupply of pharmacologically active plant-derived natural products: a review. Biotechnol Adv 33(8):1582–614. 10.1016/j.biotechadv.2015.08.001 26281720 10.1016/j.biotechadv.2015.08.001 PMC4748402

[119] Isıyel M, Ceylan H, Demir Y (2025) Bioinformatics-Based Discovery of Therapeutic Targets in Cadmium-Induced Lung Adenocarcinoma: The Role of Oxyresveratrol. Biol. Trace Elem. Res. ; 1–16. Available from: 10.1007/s12011-025-04730-x 10.1007/s12011-025-04730-x PMC12847123 40610695

[120] Bhat KPL, Kosmeder JW, Pezzuto JM (2001) Biological Effects of Resveratrol. Antioxid Redox Signal [Internet]. Dec 5 [cited 2019 Aug 27];3(6):1041–64. Available from: http://www.liebertpub.com/doi/10.1089/152308601317203567 10.1089/152308601317203567 11813979

[121] Gatz SA, Wiesmü Ller L Take a break-resveratrol in action on DNA. Carcinogenesis [Internet]. 2008 [cited 2019 Aug 22];29(2):321–32. Available from: https://academic.oup.com/carcin/article-abstract/29/2/321/2896185 10.1093/carcin/bgm276 18174251

[122] Bryant HE, Schultz N, Thomas HD, Parker KM, Flower D, Lopez E et al (2005) Specific killing of BRCA2-deficient tumours with inhibitors of poly(ADP-ribose) polymerase. Nature [Internet]. Apr 14 [cited 2018 Dec 13];434(7035):913–7. Available from: http://www.ncbi.nlm.nih.gov/pubmed/15829966 10.1038/nature03443 15829966

[123] Farmer H, McCabe H, Lord CJ, Tutt AHJ, Johnson DA, Richardson TB et al (2005) Targeting the DNA repair defect in BRCA mutant cells as a therapeutic strategy. Nature [Internet]. Apr 14 [cited 2018 Sep 27];434(7035):917–21. Available from: http://www.nature.com/articles/nature03445 10.1038/nature03445 15829967

[124] Curtin NJ (2013) Inhibiting the DNA damage response as a therapeutic manoeuvre in cancer. Br J Pharmacol 169(8):1745–1765 23682925 10.1111/bph.12244 PMC3753833

[125] Hoppe MM, Sundar R, Tan DSP, Jeyasekharan AD Biomarkers for Homologous Recombination Deficiency in Cancer. JNCI J Natl Cancer Inst [Internet]. 2018 Jul 1 [cited 2018 Dec 13];110(7):704–13. Available from: http://www.ncbi.nlm.nih.gov/pubmed/29788099 10.1093/jnci/djy085 29788099

[126] Dietlein F, Thelen L, Reinhardt HC (2014) Cancer-specific defects in DNA repair pathways as targets for personalized therapeutic approaches. Trends Genet [Internet]. Aug [cited 2018 Sep 27];30(8):326–39. Available from: http://www.ncbi.nlm.nih.gov/pubmed/25017190 10.1016/j.tig.2014.06.003 25017190

[127] Cleary JM, Aguirre AJ, Shapiro GI, D’Andrea AD (2020) Biomarker-guided development of DNA repair inhibitors. Mol Cell 18(78):1070–85 10.1016/j.molcel.2020.04.035 PMC7316088 32459988

[128] Dias MP, Moser SC, Ganesan S, Jonkers J (2021) Understanding and overcoming resistance to PARP inhibitors in cancer therapy. Nat Rev Clin Oncol 18(12):773–91. 10.1038/s41571-021-00532-x 34285417 10.1038/s41571-021-00532-x

[129] Pilié PG, Tang C, Mills GB, Yap TA (2019) State-of-the-art strategies for targeting the DNA damage response in cancer. Nat Rev Clin Oncol 16(2):81–104 30356138 10.1038/s41571-018-0114-z PMC8327299

[130] Gatz SA, Keimling M, Baumann C, Dörk T, Debatin KM, Fulda S et al (2008) Resveratrol modulates DNA double-strand break repair pathways in an ATM/ ATR-p53- and -Nbs1-dependent manner. Carcinogenesis 29(3):519–527 18174244 10.1093/carcin/bgm283

[131] Rupnik A, Lowndes NF, Grenon M (2010) MRN and the race to the break. Chromosoma 119(2):115–135 19862546 10.1007/s00412-009-0242-4

[132] Abbas T, Dutta A (2009) P21 in cancer: intricate networks and multiple activities. Nat Rev Cancer 9:400–414 19440234 10.1038/nrc2657 PMC2722839

[133] Zhang Y, Fujita N, Tsuruo T (1999) Caspase-mediated cleavage of p21(Waf1/Cip1) converts cancer cells from growth arrest to undergoing apoptosis. Oncogene 18(5):1131–1138 10022118 10.1038/sj.onc.1202426

[134] Jin YH, Yoo KJ, Lee YH, Lee SK (2000) Caspase 3-mediated cleavage of p21(WAF1/CIP1) associated with the cyclin A-cyclin-dependent kinase 2 complex is a prerequisite for apoptosis in SK-HEP-1 cells. J Biol Chem [Internet] 275(39):30256–63. 10.1074/jbc.M001902200 10884382 10.1074/jbc.M001902200

[135] Chattopadhyay D, Ghosh MK, Mal A, Harter ML (2001) Inactivation of p21 by E1A leads to the induction of apoptosis in DNA-damaged cells. J Virol 75(20):9844–9856 11559818 10.1128/JVI.75.20.9844-9856.2001 PMC114557

[136] Javelaud D, Besançon F (2002) Inactivation of p21WAF1 sensitizes cells to apoptosis via an increase of both p14ARF and p53 levels and an alteration of the Bax/Bcl-2 ratio. J Biol Chem 277(40):37949–37954 12151395 10.1074/jbc.M204497200

[137] Bellare Ganesh Pai, Patro Birija Sankar (2022) Resveratrol sensitizes breast cancer to PARP inhibitor, talazoparib through dual inhibition of AKT and autophagy flux. Biochem Pharmacol 199:115024 35367197 10.1016/j.bcp.2022.115024

[138] Sinha S, Chatterjee S, Paul S, Das B, Dash SR, Das C et al (2022) Olaparib enhances the resveratrol-mediated apoptosis in breast cancer cells by inhibiting the homologous recombination repair pathway. Exp Cell Res 420(1):113338. 10.1016/j.yexcr.2022.113338 36075449 10.1016/j.yexcr.2022.113338

[139] Hu C, Liu Y, Teng M, Jiao K, Zhen J, Wu M et al (2019) Resveratrol inhibits the proliferation of estrogen receptor-positive breast cancer cells by suppressing EZH2 through the modulation of ERK1/2 signaling. Cell Biol Toxicol 35(5):445–456 30941654 10.1007/s10565-019-09471-x

[140] Guo X, Zhao Z, Chen D, Qiao M, Wan F, Cun D et al (2019) Co-delivery of resveratrol and docetaxel via polymeric micelles to improve the treatment of drug-resistant tumors. Asian J Pharm Sci 14(1):78–85. 10.1016/j.ajps.2018.03.002 32104440 10.1016/j.ajps.2018.03.002 PMC7032195

[141] Berardi D, Hunter Y, van den Driest L, Farrell G, Rattray NJW, Rattray Z (2022) The differential metabolic signature of breast cancer cellular response to Olaparib treatment. Cancers (Basel) 14(15):1–37 10.3390/cancers14153661 PMC9367310 35954325

[142] Keung MY, Wu Y, Badar F, Vadgama JV (2020) Response of breast cancer cells to PARP inhibitors is independent of BRCA status. J Clin Med 9(4):940 32235451 10.3390/jcm9040940 PMC7231148

[143] Gilardini Montani MS, Prodosmo A, Stagni V, Merli D, Monteonofrio L, Gatti V et al (2013) ATM-depletion in breast cancer cells confers sensitivity to PARP inhibition. J Exp Clin Cancer Res 32(1):1–10 24252502 10.1186/1756-9966-32-95 PMC4176289

[144] Sinha S, Paul S, Acharya SS, Das C, Dash SR, Bhal S et al (2024) Combination of resveratrol and PARP inhibitor Olaparib efficiently deregulates homologous recombination repair pathway in breast cancer cells through inhibition of TIP60-mediated chromatin relaxation. Med Oncol 41(2):1–18. 10.1007/s12032-023-02279-0 10.1007/s12032-023-02279-0 38184505

